# Ecology of aerobic anoxygenic phototrophs on a fine-scale taxonomic resolution in Adriatic Sea unravelled by unsupervised neural network

**DOI:** 10.1186/s40793-024-00573-6

**Published:** 2024-04-29

**Authors:** Iva Stojan, Danijela Šantić, Cristian Villena-Alemany, Željka Trumbić, Frano Matić, Ana Vrdoljak Tomaš, Ivana Lepen Pleić, Kasia Piwosz, Grozdan Kušpilić, Živana Ninčević Gladan, Stefanija Šestanović, Mladen Šolić

**Affiliations:** 1 Institute of Oceanography and Fisheries, Šetalište Ivana Meštrovića 63, Split, Croatia; 2 Doctoral Study of Biophysics, Faculty of Science, University of Split, Ruđera Boškovića 37, Split, Croatia; 3 Laboratory of Anoxygenic Phototrophs, Institute of Microbiology, Czech Academy of Sciences, 379 81 Třeboň, Czechia; 4 Department of Ecosystem Biology, Faculty of Science, University of South Bohemia, České Budějovice, Czechia; 5 University Department of Marine Studies, University of Split, Ruđera Boškovića 37, Split, Croatia; 6 Department of Fisheries, Oceanography and Marine Ecology, National Marine Fisheries Research Institute, Gdynia, Poland

## Abstract

**Background:**

Aerobic anoxygenic phototrophs are metabolically highly active, diverse and widespread polyphyletic members of bacterioplankton whose photoheterotrophic capabilities shifted the paradigm about simplicity of the microbial food chain. Despite their considerable contribution to the transformation of organic matter in marine environments, relatively little is still known about their community structure and ecology at fine-scale taxonomic resolution. Up to date, there is no comprehensive (*i.e.* qualitative and quantitative) analysis of their community composition in the Adriatic Sea.

**Results:**

Analysis was based on *puf*M gene metabarcoding and quantitative FISH-IR approach with the use of artificial neural network. Significant seasonality was observed with regards to absolute abundances (maximum average abundances in spring 2.136 ± 0.081 × 10^4^ cells mL^−1^, minimum in summer 0.86 × 10^4^ cells mL^−1^), FISH-IR groups (*Roseobacter* clade prevalent in autumn, other Alpha- and Gammaproteobacteria in summer) and *puf*M sequencing data agglomerated at genus-level. FISH-IR results revealed heterogeneity with the highest average relative contribution of AAPs assigned to *Roseobacter* clade (37.66%), followed by Gammaproteobacteria (35.25%) and general Alphaproteobacteria (31.15%). Community composition obtained via* puf*M sequencing was dominated by Gammaproteobacteria clade NOR5/OM60, specifically genus *Luminiphilus*, with numerous rare genera present in relative abundances below 1%. The use of artificial neural network connected this community to biotic (heterotrophic bacteria, HNA and LNA bacteria, *Synechococcus, Prochlorococcus,* picoeukaryotes*,* heterotrophic nanoflagellates, bacterial production) and abiotic environmental factors (temperature, salinity, chlorophyll *a* and nitrate, nitrite, ammonia, total nitrogen, silicate, and orthophosphate concentration). A type of neural network, neural gas analysis at order-, genus- and ASV-level, resulted in five distinct best matching units (representing particular environments) and revealed that high diversity was generally independent of temperature, salinity, and trophic status of the environment, indicating a potentially dissimilar behaviour of aerobic anoxygenic phototrophs compared to the general bacterioplankton.

**Conclusion:**

This research represents the first comprehensive analysis of aerobic anoxygenic phototrophs in the Adriatic Sea on a trophic gradient during a year-round period. This study is also one of the first reports of their genus-level ecology linked to biotic and abiotic environmental factors revealed by unsupervised neural network algorithm, paving the way for further research of substantial contribution of this important bacterial functional group to marine ecosystems.

**Supplementary Information:**

The online version contains supplementary material available at 10.1186/s40793-024-00573-6.

## Background

Aerobic anoxygenic phototrophs (AAPs) are a polyphyletic group of bacteria capable of photoheterotrophy. They are omnipresent in diverse habitats, and were discovered in the 1970s [1]. In recent decades, they have been recognised as an important functional group of particular interest in microbial ecology, and extensive studies have been conducted on this topic [2–10]. AAPs are considered ubiquitous in the marine environment with first quantitative estimates of their abundances up to 11% of the total microbial community in the upper open ocean [3]. A recent study reports their record abundance in the Adriatic Sea with up to 30% of the bacterial population in the piconeuston community following open fire events [11].

AAPs are facultative photoheterotrophs that harvest light energy and generate ATP by photophosphorylation using a unique type of bacteriochlorophyll-*a*-containing reaction center. Nevertheless, they primarily rely on dissolved organic matter as an energy source [12]. They exhibit higher growth rates and larger cell volumes compared to other bacterioplankton, making them particularly vulnerable to predation [13–16]. Hence, their contribution to the transformation of both organic and inorganic matter in aquatic environments is substantial [5, 8, 10, 17]. Ecology of AAPs in different ecosystems is rather complex, not yet fully understood, and influenced by a plethora of factors, such as temperature, salinity, nutrient availability, light intensity, and the presence of predators [5, 7, 9, 14, 16]. Furthermore, the abundance and distribution of AAPs has been shown to vary significantly on a spatiotemporal scale with distinct seasonality and could be regulated by ocean circulation patterns and seasonal changes in sunlight availability [5, 9].

Based on the metagenomic approach and metabarcoding of the *puf*M gene, marine AAPs are so far taxonomically classified into the proteobacterial classes Alpha- and Gammaproteobacteria [5, 18, 19]. Sequencing amplicons of the *puf*M gene, encoding the M-chain of the photosynthetic reaction centre complex, is currently the most common approach in the investigation of their ecology. This preference stems from the realisation that metagenomic approach may overlook certain groups that occur at very low abundances [5]. In comparison with sequencing-based methodologies which do not provide quantitative estimates of specific taxa [20], infrared epifluorescence microscopy combined with fluorescence in situ hybridisation (FISH-IR) aims for quantification of specific AAP groups [21]. Therefore, a combination of qualitative and quantitative methodologies is required for a comprehensive analysis.

Even though various studies have been conducted over the years on the topic of AAP abundance, distribution, ecology, and dynamics [11, 22–25], their community composition remains unknown in the Adriatic Sea. Hence, here we present the first comprehensive analysis of this community in the Adriatic Sea along a trophic gradient during a year-round period. This study: (i) elucidates patterns of AAP distribution and community composition based on *puf*M metabarcoding in the central Adriatic, (ii) quantifies abundances of the main AAP groups with FISH-IR, (iii) reveals biotic and abiotic environmental factors potentially affecting AAPs on a fine-scale taxonomic resolution using a neural gas algorithm, ultimately broadening our knowledge of their composition and ecology.

## Methods

### Study area, environmental parameters and plankton analysis

**Fig. 1:**
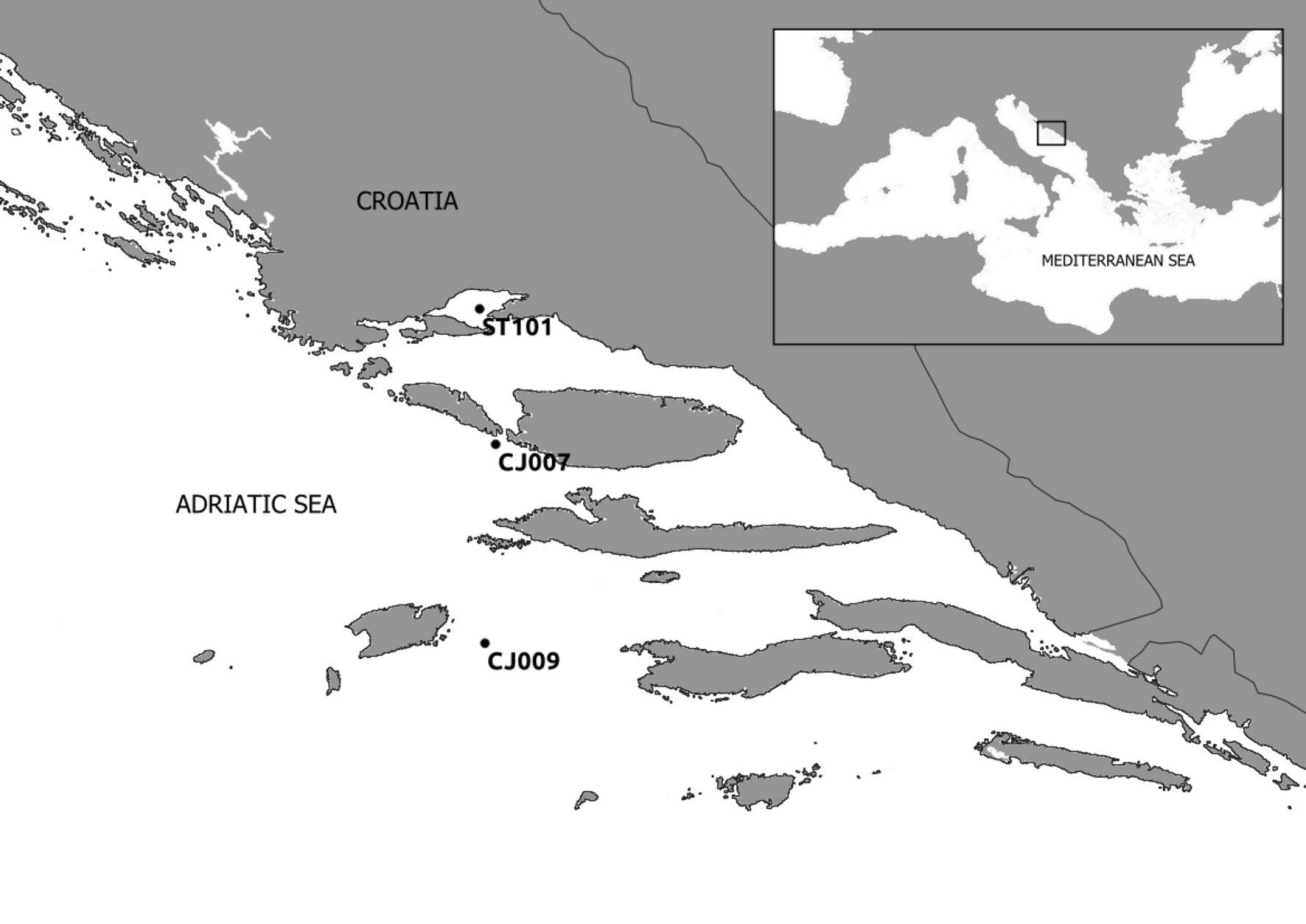
Study area of the central Adriatic Sea with sampling stations ST101, CJ007 and CJ009

A total of 90 samples (Additional file 1: Table S1) were collected on board the R/V BIOS DVA, predominantly on a monthly basis (with the exception of July, September and October) from February 2021 to January 2022 on vertical profiles at three stations in the central Adriatic Sea: the coastal area ST101 (0 m and 35 m depths), the channel CJ007 (0 m, 30 m and 50 m depths) and the open sea CJ009 [0 m, 30 m, 50 m, 75 m, 100 m and deep chlorophyll maximum (dChlMax) depths] (Fig. 1).

**Fig. 2:**
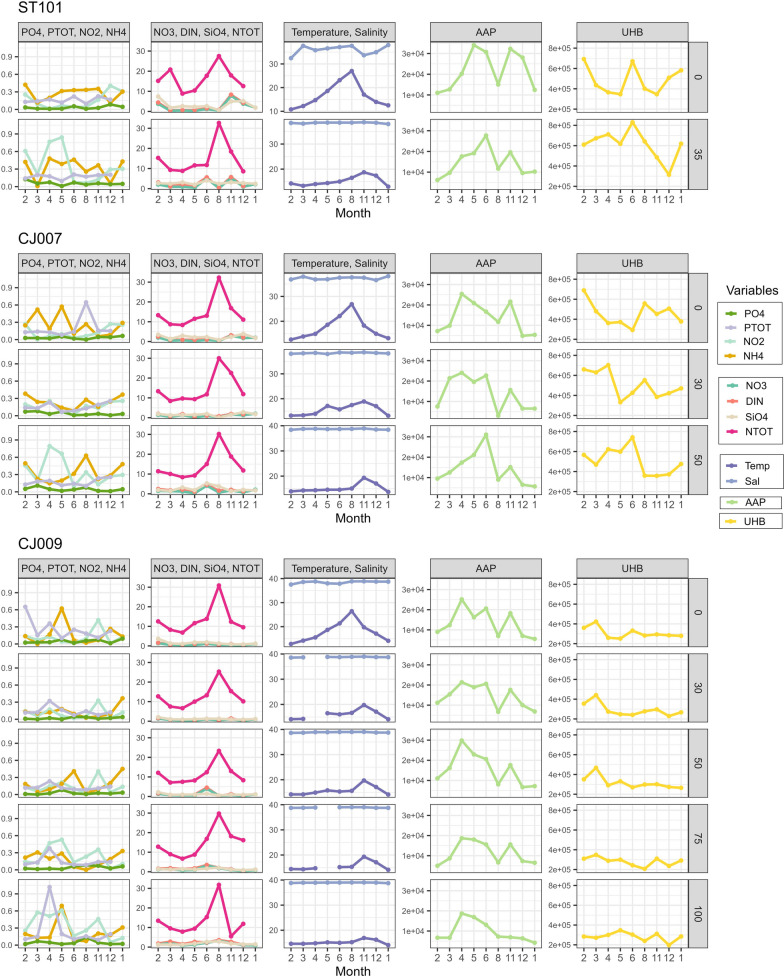
Abiotic variables (temperature-Temp, salinity-Sal, nitrate- NO_3_^−^, nitrite- NO_2_^−^, ammonium ion- NH_4_^+^, dissolved inorganic nitrogen-DIN, total nitrogen-NTOT, orthophosphate- PO_4_^3−^, total phosphorus-PTOT, silicate- SiO_4_^2−^) with absolute abundances of total heterotrophic bacteria (UHB) and aerobic anoxygenic phototrophs (AAPs) shown per station, month and depth

Seasons were determined according to the criteria of a comprehensive historical hydrographic dataset of the Adriatic Sea, with the months January to April being considered winter, May and June spring, July to October summer and November and December autumn [26]. Temperature, salinity, nutrients concentrations and chlorophyll *a* (Chl *a*) were measured as described in a previous study [27] and values are shown in Fig. 2. Abundances of picoplankton community members, namely *Synechococcus* and *Prochlorococcus*, picoeukaryotes (PE), heterotrophic bacteria, high and low nucleic acid bacteria (HNA and LNA bacteria, respectively) and heterotrophic nanoflagellates (HNF), were measured by flow cytometry as previously described [27]. Bacterial production was estimated by the 3H-thymidine incorporation method as previously described [28].

### Aerobic anoxygenic phototrophs (AAPs) abundance

Seawater samples for epifluorescent microscopy were collected using a Niskin bottle (5 L). After immediate fixation of 50 mL aliquotes with formaldehyde (pH 7.5, final concentration (f. c.) 2%) for 1 h at room temperature or overnight at 4 °C, samples were filtered on white 0.2 µm polycarbonate filters (47 mm diameter, Whatman® Nuclepore™ Track-Etched, Merck) and, after drying, stained with 4’,6-diamidino-2-phenylindole (DAPI, f. c. 1 μgmL^−1^) using a 3:1 mixture of Citifluor™ AF1 and Vectashield® [29]. AAP bacteria were counted using an Olympus BX51 microscope equipped with an Olympus UPlanSApo 100 × /1.40 OIL, infra-red (IR) objective, a U-LH100H6 Hg lamp for excitation and image analysis software (CellSens). Images were taken with an XM10- IR camera (Olympus). Due to the rapid fading of the bacteriochlorophyll-*a* (Bchl *a*) autofluorescence, three epifluorescent filter sets were applied in a specific order: IR, DAPI and Chl *a*. The Chl *a* signal was subtracted from IR to obtain a net count of AAP cells.

### Infrared epifluorescence microscopy and fluorescence in situ hybridisation (FISH-IR)

A combination of two epifluorescence-based methods, infrared epifluorescence microscopy and fluorescence in situ hybridisation (FISH-IR) was applied to simultaneously detect infrared and probe signals, as described previously [21, 29, 30]. Probes targeting Alphaproteobacteria (probe ALF968), Gammaproteobacteria (probe GAM42a and unlabelled Bet42a competitor) and the *Roseobacter* clade (probe ROS537) were used. For detailed protocol, please see Additional file 2: Methods.

### DNA extraction, *pufM* amplification, and Illumina amplicon sequencing

Seawater for DNA analyses was collected using a Niskin bottle (5 L), pre-filtered through a 20-µm plankton net and 1–2 L were immediately vacuum filtered on board through 0.22-µm polyethersulfone membrane filters (PES, 47 mm diameter, FiltraTECH, France). One liter of Milli-Q water represented a negative filtration control. Filters were frozen in liquid nitrogen and stored at − 80 °C until further analyses. A modified DNeasy PowerWater kit (QIAGEN, The Netherlands) with an enhanced bead-beating step was used for DNA extraction [31]. Negative control (extraction blank) included empty 0.22-µm polyethersulfone membrane filter (PES, 47 mm diameter, FiltraTECH, France). Briefly, PES filters were cut in half with a sterilised scalpel and cut into smaller pieces, placed in 1.5 mL tubes filled with ceramic beads (MagNA Lyser Green Beads, Roche, Switzerland), followed by rigorous homogenisation with the MagNALyser instrument, twice for 20 s at 9000 RCF. After homogenisation, the tubes were centrifuged at 5600 RCF for 1 min. The supernatant was transferred to a clean collection tube and the protocol was performed according to the manufacturer’s instructions from step 8 in the Quick Start Protocol. The DNA was finally eluted in 35 µL of the EB solution. Total extracted DNA was quantified and A260/A280 and A260/A230 absorbance ratios were measured using DS -11 spectrophotometer (Denovix, USA). Negative filtration and extraction controls showed DNA concentrations below the limit of detection. To analyse the composition of the AAP community, amplification of *puf*M gene (~ 204 bp) was performed with pufM_UniF (5′-GGNAAYYTNTWYTAYAAYCCNTTYCA) and pufM_WAW (5′-AYNGCRAACCACCANGCCCA) primers [16, 32, 33]. All samples were amplified in triplicate, with 25 µL of reaction mix each containing 12.5 µL of Q5® High-Fidelity 2X Master Mix (New England Biolabs, USA), 1.25 µL of each primer at a final concentration of 0.5 µM, 2 µL of DNA template (10 ng/µL, final mass of DNA template in PCR reaction 20 ng) and 8 µL of sterile, nuclease-free water. Cycling conditions were as follows: initial denaturation at 98 °C for 30 s, followed by 35 cycles of amplification at 98 °C for 7 s, 58 °C for 30 s and 72 °C for 30 s, with 2 min of final extension at 72 °C (T100 thermal cycler, Biorad, USA). The triplicates were purified from the agarose gel (2.0%) and pooled using the Wizzard SV Gel and PCR Clean-Up System (Promega, USA) according to the manufacturer’s instructions and then quantified using the DS -11 spectrophotometer (Denovix, USA). All filtration/extraction blanks and PCR non-template controls were negative. All DNA extractions and PCR amplifications were performed in the same laboratory by the same person. Library preparation and amplicon pair-end sequencing (2 × 250 bp) on the Illumina MiSeq were performed by the Genomics Core Facility of the Universitat Pompeu Fabra, Barcelona, Spain.

### Bioinformatics, phylogenetic placement and calculation of diversity and evenness

A total of 7,823,083 input reads from Illumina Miseq were used for the bioinformatic analyses. The initial counts per sample are given in Additional file 1: Table S2. The quality of raw forward and reverse reads was assessed with FastQC v0.11.9. Primers were trimmed using cutadapt v4.1 [34]. Subsequent data processing was performed with the statistical software R v4.0.2 (https://cran.r-project.org/) using the R package DADA2 v1.16.0 [35]. Briefly, *filterAndTrim* function (truncLen = c(200, 200), maxN = 0, maxEE = c (2, 2), truncQ = 2, rm.phix = TRUE) removed low quality sequences and sequence tails. After error learning and sample inference algorithm, the paired-end reads were merged, resulting in the amplicon sequence variant (ASV) table. The *removeBimeraDenovo* function with "pooled" method discarded chimeras that accounted for 5.8% of the merged sequence reads. The naive Bayesian classification method [36] was used to assign taxonomy with the *assignTaxonomy* function based on the manually curated database, the most complete for AAPs to date [6].

Due to the high proportion of reads unclassified at genus level in the taxonomic assignment using DADA2, as high as 50% in some samples, a phylogenetic analysis was performed. ASVs occurring less than 2 times in at least 5% of samples and samples with an unacceptably low final number of reads per sample (N < 2000) were excluded from further analysis using the R package phyloseq v1.32.0 [37], resulting in 663 unique ASVs (number of reads per sample: median 58,227, min 2,039, max 32,5210) in 81 samples. The 663 ASVs were aligned using MAFFT [38] and 2 ASVs that poorly aligned were removed and further analysis was performed using 661 ASVs. A database of 3363 *puf*M sequences and their taxonomic assignment [6] was used for taxonomic assignment of the ASVs. ASVs were placed in the *puf*M gene phylogenetic tree [6] using the Evolutionary Placement Algorithm v0.3.5 [39] and Gappa [40] handled the taxonomic assignment of ASVs according to their position in the phylogenetic *puf*M tree with a consensus threshold higher than 50%. Sample data, untransformed final ASV matrix, taxonomy and reference *puf*M sequences (refseq) are given in Additional file 3. Since most of the initial *puf*M phylogroups [41] have already been assigned to a standard phylogenetic taxonomy [19], just the Rhodobacterales ASVs which could potentially belong to distinct *puf*M phylogroups E, F or G were aligned with phylogroup sequences using MAFFT [38]. A phylogenetic tree was built using FastTree [42] to obtain phylogroup affiliation or to facilitate the comparison with previous publications that used phylogroup taxonomic affiliation [5, 19, 41].

The R package ggplot2 v3.3.5 [43] was used to graphically visualize the composition of the AAP community in terms of relative abundances of a given taxon at class, order and genus levels. For agglomeration of ASVs to a certain taxonomic rank (order or genus); function *tax_glom* from R package phyloseq v1.32.0 was used. Since microbiome count data is of a compositional nature and should be treated as such [20, 44, 45], centered log-ratio (CLR) transformation [46] was performed using function *transform* from R package microbiome v1.10.0 [47], with introduced pseudo-counts (minimum relative abundance divided by two) instead of zeros in ASV table before the transformation.

In order to account for significant discrepancies in read counts between samples, rarefying (*i.e.* random subsample of reads to equalise read depth) was used exclusively to estimate the diversity metrics. Rarefying to the smallest library size multiple times (function *rarefy_even_depth* from phyloseq v1.32.0 R package, rarefying threshold = 2000, N(times) = 100) was applied on the ASV matrix to estimate alpha diversity metrics calculated as mean values of multiple subsample calculations: the observed number of ASVs, Shannon's (H') and Pielou's (J') indices calculated with vegan v2.5.7 R package [48]. Rarefaction curve exhibiting sufficient sequencing depth for diversity estimates is given in Additional file 1: Fig. S1.

### Statistical analyses and neural gas algorithms

Correlations between abiotic environmental variables were assessed using correlational (Draftsman) plots in PRIMER7 to estimate if a certain variable should be excluded in the case of a strong correlation (Additional file 1: Fig. S2). No variables were excluded from further analysis.

To assess whether there are differences in the spatiotemporal patterns of the AAP community, permutational multivariate analysis of variance (PERMANOVA) based on Aitchison distances (Euclidean distances on CLR transformed dataset) was performed on the *puf*M dataset in PRIMER7 software, with 'season' and 'region' as fixed factors and 'layer' as a random factor nested in 'region' (9999 permutations, sums of squares type: type II (conditional), permutation method: Unrestricted permutation of raw data) [49, 50]. Factor layer was defined as L1 (0–30 m), L2 (30–50 m), L3 (50–75 m) and L4 (75–100 m). Accompanying PERMANOVA, distance-based test for homogeneity of multivariate dispersions (PERMDISP) was performed on the same transformed dataset (9999 permutations, deviations from centroid). A significant result of PERMDISP would indicate that groups differ in dispersion [51]. Variance-based compositional principal component (PCA) biplot on Aitchison distances was generated based on genus-level agglomerated and CLR-transformed values with zero replacement using pseudo-counts with the R package microViz v0.10 [52].

AAP absolute and relative abundance data as well as FISH-IR microscopic counts given as relative abundances were square root transformed. Non-metric multidimensional scaling (nMDS) ordination plot based on Bray–Curtis distances was constructed to visualise differences in seasonal abundances for FISH-IR groups, followed by PERMANOVA to test the significance of observed differences (9999 permutations, sums-of-squares type: type II (conditional), permutation method: Unrestricted permutation of raw data). PERMDISP (9999 permutations, deviations from centroid) was performed accompanying PERMANOVA on the same transformed dataset. To assess agreement between metabarcoding and FISH-IR data, Spearman correlation coefficients and their statistical significance were calculated between FISH-IR probe counts and corresponding pseudoabundances from *puf*M sequencing (*i.e*. relative abundance of Gammaproteobacteria class × AAP absolute number/100) using *cor.test* function from R package stats v.3.6.2 (Additional file 4).

An artificial neural network/unsupervised topology learning algorithm, neural gas (NG), was applied to a CLR-transformed *puf*M dataset to estimate characteristic AAP patterns associated with specific biotic/abiotic environmental factors [53]. NG has been successfully used for this type of data in previous studies due to its suitability for modelling anomalies and the mean distribution of microbiological parameters [25, 27, 54]. In our study, separate NG models were created on agglomerated and CLR-transformed datasets on *puf*M sequencing data, carried out at the order-, genus- and ASV-levels. NG models used the *puf*M sequencing data for quantifying data space to generate "best-match units" (BMUs). For all models, the ecological factors (biotic: heterotrophic bacteria-UHB, High nucleic acid bacteria-HIGH, *Synechococcus*-SYN, *Prochlorococcus*-PROCHL, picoeukaryotes-PE, bacterial production-BP, hetrotrophic nanoflagellates-HNF, aerobic anoxygenic phototrophs-AAP; and abiotic: temperature-Temp, salinity-Sal, nitrates-NO_3_^−^, nitrites-NO_2_^−^, ammonium ion-NH_4_^+^, dissolved inorganic nitrogen-DIN, total nitrogen-NTOT, soluble reactive phosphorus-SRP, total phosphorus-PTOT, silicate-SiO_4_^2−^, Chlorophyll *a*-Chl *a*) were calculated as average values for a specific BMU. All models resulted in five characteristic distributions (BMUs). The models were initialised by setting the number of training epochs to 1000, the initial step size to 0.5 and the initial decay constant to 4.5 using SOM Toolbox version 2.0 for MATLAB (E. Alhoniemi, J. Himberg, J. Parhankangas and J. Vesanto, Helsinki University of Technology, Finland: http://www.cis.hut.fi/projects/somtoolbox).

Heatmap presentation of taxa in each BMU was generated using conditional formatting in Microsoft Excel v.16.0: values of environmental variables were coloured according to their average values for every BMU, and taxa according to their average CLR-transformed values. Further, *heatmap.2* function of gplots v3.1.3 R package was used to construct heatmaps representing community composition based on Aitchison distances and Ward.D2 dendrogram agglomeration method [55].

## Results

### Abundance of AAP bacteria

AAPs were observed in all 90 samples (Fig. 2) and their mean absolute abundance in the study area was 1.43 ± 0.75 × 10^4^ cells mL^−1^. The lowest value was recorded at station CJ007 in August at a depth of 30 m (0.30 × 10^4^ cells mL^−1^), while the highest value was observed at station ST101 in May at the sea surface (3.41 × 10^4^ cells mL^−1^). The average relative abundance was 3.86% ± 2.27% (minimum 0.55% at station CJ007 in August at 30 m depth; maximum 10.26% at CJ009 in April at 50 m depth). At the monthly level, the highest mean abundance was recorded in June with 2.19 ± 0.62 × 10^4^ cells mL^−1^, followed by almost identical values in April with 2.18 ± 0.41 × 10^4^ cells mL^−1^, while the lowest mean abundance was observed in January 2022 with 0.7 ± 0.25 × 10^4^ cells mL^−1^ and in February 2021 with 0.84 ± 0.22 × 10^4^ cells mL^−1^.

**Fig. 3:**
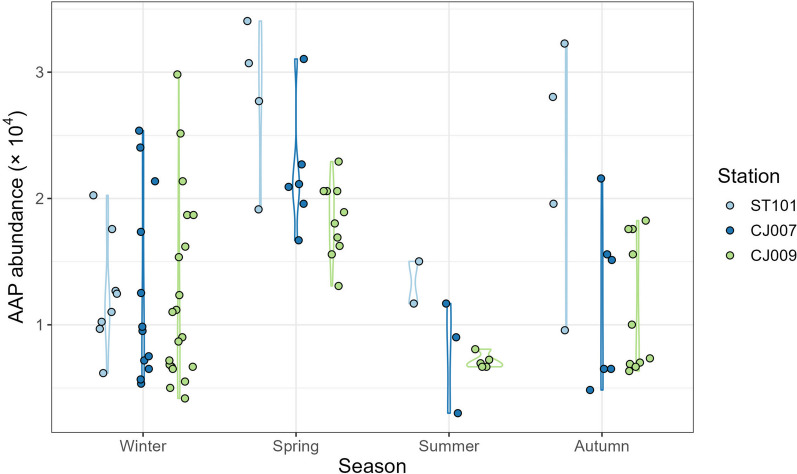
Absolute abundances of AAPs shown per station (ST101, CJ007 and CJ009) and season

Absolute and relative abundances of AAPs significantly differed on a seasonal scale (PERMANOVA pseudo-F = 23.13, *p* = 0.0001, unique permutations = 9957; pseudo-F = 27.98, p = 0.0001, unique permutations = 9948 respectively), but not with respect to region or depth (Additional file 1: Table S3A and S3B respectively), although a decreasing trend was observed towards the open sea (Fig. 3). The pairwise comparisons showed that spring was the only season different from others (Additional file 1: Table S3). However, seasonal absolute abundances also differed in dispersion (PERMDISP test, group factor: Season, F = 5.05, p = 0.0067, 9999 permutations), while relative abundances did not (PERMDISP test, group factor: Season, F = 1.51, p = 0.27), suggesting differences also stem from larger variances between samples.

### FISH-IR

To quantify which proportion of AAPs belong to the Alphaproteobacteria and Gammaproteobacteria classes or to the *Roseobacter* clade, a method based on epifluorescence microscopy, FISH-IR, was used. In the study area, gammaprotebacterial AAPs had a mean relative abundance of 35.25%, Alphaproteobacteria 31.15% and *Roseobacter* 37.66%. Complete results are presented and visualized in Additional file 2. During data acquisition and processing, several limitations of the ALF968 probe, targeting all Alphaproteobacteria were observed: percentage of AAP cells hybridized with the ROS537 probe (*Roseobacter* clade within Alphaproteobacteria) generally exceeded the number of cells detected with the ALF968 probe, and a total sum of all probes was often greater than 100%. Therefore, in silico coverage and specificity of ALF968 and ROS537 probes for the order Rhodobacterales were estimated against SILVA database. The ALF968 had larger coverage than the ROS537 probe (96.3% vs. 92.9%), but showed reduced specificity (92.96% vs. 99.32% respectively), which might explain this phenomenon (Additional file 5). After correlating FISH-IR and *puf*M metabarcoding data, we observed strong positive correlation (r = 0.745) for Gammaproteobacteria, moderate positive correlation (r = 0.651) for Alphaproteobacteria and strong positive correlation (r = 0.717) for *Roseobacter,* indicating consistency in the relative abundance patterns between these two methods. All correlations were statistically significant (p < 0.001) (Additional file 4). We conclude that results from FISH-IR probes Gam42a and ROS537 targeting class Gammaproteobacteria and *Roseobacter* clade respectively can be trusted and contribute additional value to data quantitative interpretation.

AAPs belonging to *Roseobacter* clade showed the highest relative abundance (77.77%) at station ST101 at 35 m in August and the lowest one (14.29%) at CJ007 station at 50 m in August. The highest average relative abundances were recorded in autumn and the lowest average ones in summer, except for ST101 station (Additional file 2: Figs. S2 and S3). Gammaproteobacteria AAPs had the highest relative abundances in samples collected from the surface (0 m) of open sea station CJ009 in February (78.9%), followed by samples from station CJ007 at 30 m depth in August (75%) and station ST101 at 35 m depth also in August (71.4%). The lowest value (8.3%) was measured at the sea surface in January at station CJ009. In terms of seasonal distribution, they were on average predominant in summer at all stations, with the lowest contribution in winter (Additional file 2: Figs. S2 and S3).

### AAP community composition on a spatiotemporal scale obtained via metabarcoding

**Fig. 4:**
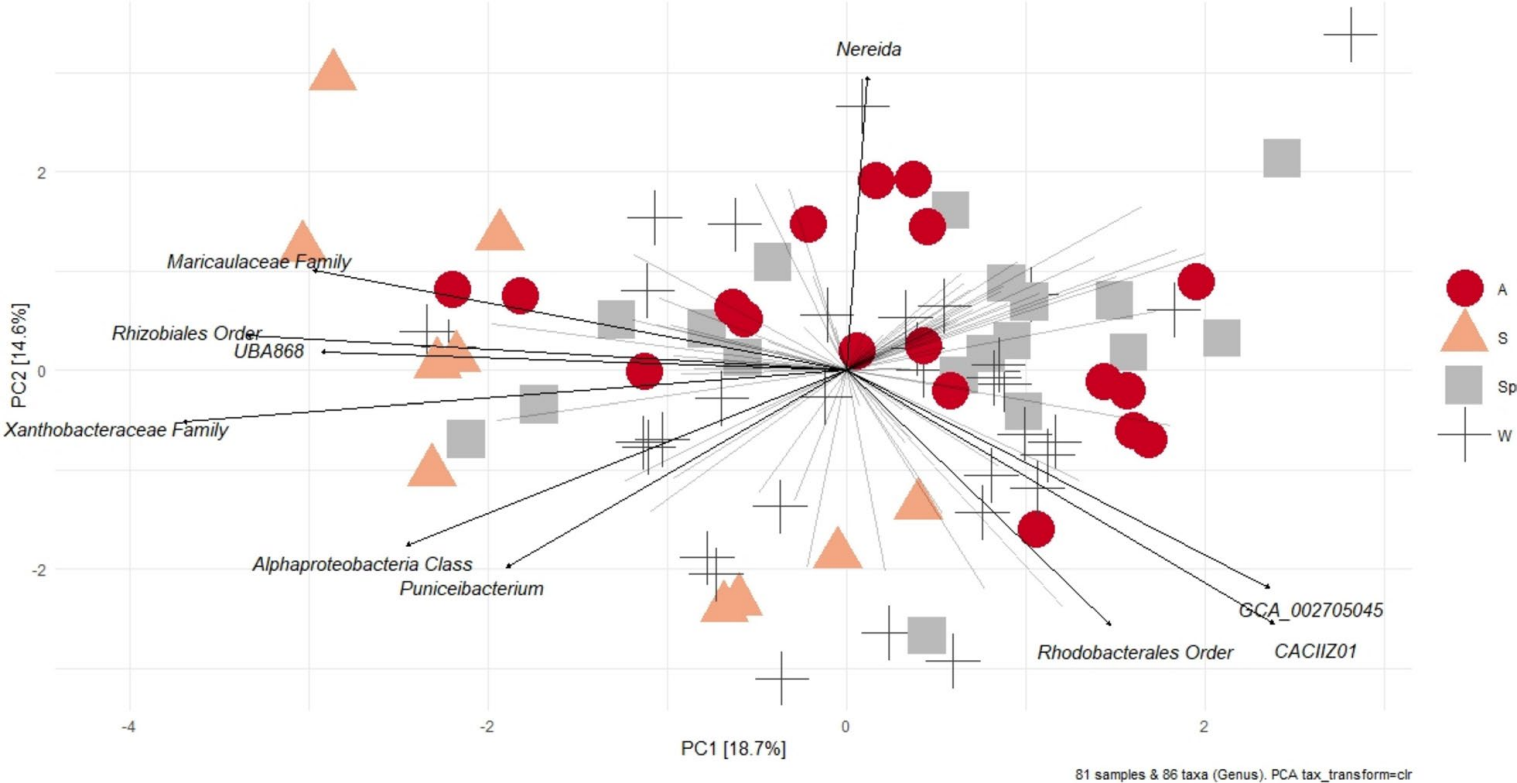
Variance-based compositional principal component (PCA) biplot on Aitchison distances of *puf*M dataset (agglomerated at genus-level and CLR transformed) showing groupings by season (W-Winter, Sp-Spring, S-Summer, A-Autumn) where names of top 10 taxa by the longest loading vector length are indicated

When considering the composition of the AAP community obtained from the *puf*M sequencing dataset agglomerated at the genus level, statistically significant seasonal differences were observed (PERMANOVA, pseudo-F = 2.06, p = 0.0037, unique permutations = 9890, Additional file 1: Table S4). Similarly to total AAP abundances, there were no differences between spatial or vertical profiles. Pairwise comparisons showed that summer differed from other seasons (Additional file 1: Table S4). There were no differences in dispersion between seasons (PERMDISP, F = 1.89, p = 0.2035, 9999 permutations). Slight separation of summer samples from others was observed in PCA biplot, with possible subdivision within the group (Fig. 4). Taxa contributing most to separation of summer from other seasons (with higher contribution during summer) were *Puniceibacterium* genus*,* UBA868, Xanthobacteraceae and Maricaulaceae family (Fig. 4).

Phylum Proteobacteria dominated in all samples. Interestingly, the phylum Gemmatimonadota, family Gemmatimonadaceae, represented by two ASVs (ASV550 and ASV559) unclassified at genus level, was observed in a very low relative abundance (mean 0.002%) in a sample collected in April at 30 m depth at station CJ007, as well as in few others in a very low number of reads. For details, please see Additional file 3 (sheets "ASV_matrix" and "Taxonomy").

At the class level, the AAP community composition was dominated by Gammaproteobacteria in all samples (75.9% on average), with the highest values in December at stations ST101 and CJ007 and in November at station CJ009. In contrast, the lowest relative contribution was recorded in February at the sea surface at station ST101, when their relative abundance dropped < 40%, and in March, also at the sea surface at station CJ007 with relative abundances of ~ 50% (Additional file 1: Fig. S3). The alphaproteobacterial AAPs had an average relative abundance across all samples of 24.1% with the highest contribution to the total number of reads among the sea surface samples in February at ST101 (~ 60%) and in March at CJ007 (~ 40%).

**Fig. 5:**
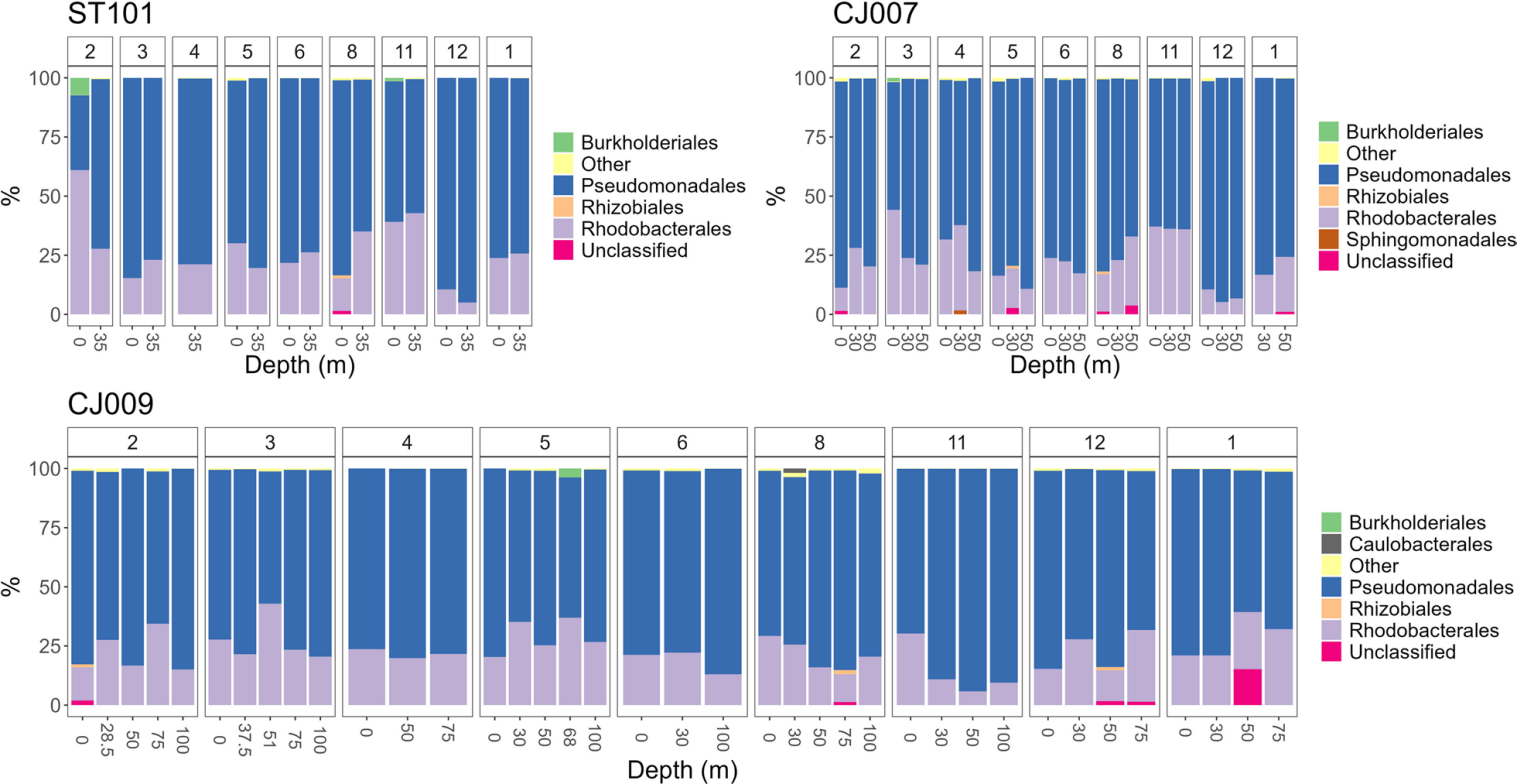
AAP orders detected in the study area via *puf*M metabarcoding shown per station (ST101, CJ007 and CJ009), month and depth

Looking at AAP orders, the gammaproteobacterial Pseudomonadales dominated at all stations and in all months during the sampling period (Fig. 5), except for the lowest contribution in February at the sea surface at station ST101 (< 40%) and in March, also at the sea surface at station CJ007 (~ 50%). Burkholderiales had higher relative abundance (~ 10%) in February at 0 m at station ST101 and in May at dChlMax (non-standard oceanographic depths, 68 m) at station CJ009. Order Rhodobacterales (family Rhodobacteraceae), which represented 96% of Alphaproteobacteria, was also omnipresent at all stations and in all months, with the highest relative contribution recorded in February at the sea surface at station ST101 (~ 60%) and in March, also at the sea surface at station CJ007 (~ 40%). In contrast, the alphaproteobacterial orders Rhizobiales, Sphingomondales and Caulobacterales occurred occasionally in relative abundances > 1% (Fig. 5).

**Fig. 6:**
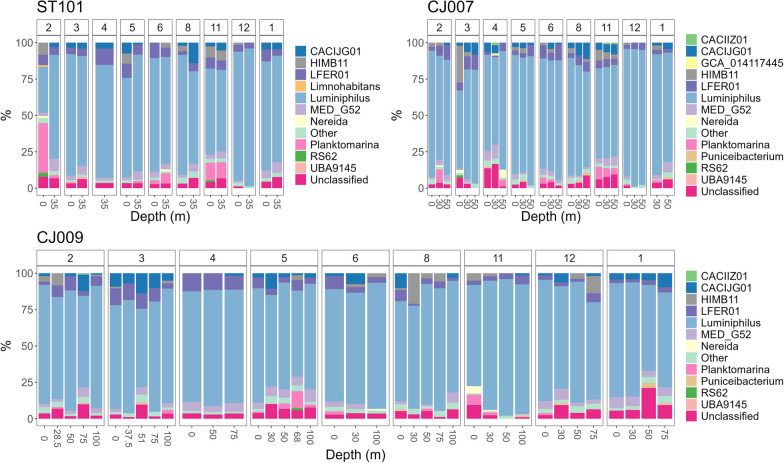
AAP community composition via* puf*M metabarcoding in the study area at the genus level shown per station (ST101, CJ007, CJ009), month and depth

Numerous AAP genera (71 of total 86) occurred in relative abundances of less than 1%, while the 15 most prevalent ones accounted for 99% of the sequencing data (Fig. 6). In general, genus *Luminiphilus* (phylogroup K, order Pseudomonadales, family Halieaceae) dominated the AAP community, with relative abundance exceeding 90% in some samples. In total, 146 different ASVs were recorded for this genus, with different relative contribution at specific stations, depths and months. Details about the distribution of *Luminiphilus* ASVs are given in Additional file 6. *Limnohabitans* was detected in abundance > 1% at sea surface station ST101 in February, but not towards the open sea. The Alphaproteobacteria *CACIIZ01* and Rhodobacteraceae *Puniceibacterium* were not recorded at costal station ST101, and sporadically on other stations (Fig. 6). The genus *Planktomarina* showed the highest relative abundance (~ 20%) at station ST101 in February at the sea-surface and higher values in November regardless of depth at stations ST101 and CJ007. Genus *MED_G52* of the Rhodobacteraceae family was present in abundance up to 10% at all stations, in all seasons and depths, except in December at stations ST101 and CJ007 (present in > 1% only at 0 m) and in November at CJ009 (< 1%).

The lowest average number of observed ASVs was recorded in winter (124.34 ± 50.9), while the highest one was recorded in autumn (132.6 ± 56.38). The average Shannon diversity indices were highest in summer (3.5 ± 0.44) and lowest in autumn (3.39 ± 0.68), while the average values for winter and spring months were in between (3.45 ± 0.39 and 3.47 ± 0.35, respectively). The average Pielou’s evenness showed the same trend as Shannon indices, with the highest average values in summer (0.73 ± 0.05) and the lowest in autumn (0.70 ± 0.08) (Additional file 1: Table S5).

### Neural gas analyses of *pufM* gene metabarcoding data

To gain a deeper insight into AAP community composition at a finer taxonomic resolution and their structuring in the light of biotic and environmental parameters, neural gas (NG) analysis, an artificial neural network algorithm robust to outliers, was applied to *puf*M sequencing dataset at order-, genus- and ASV-levels. Agglomeration of dataset to higher taxonomic rank (order) was necessary for comparison to previous literature, and the rationale behind running multiple analyses on lower taxonomic ranks was to reveal if a particular genus within order, or ASVs of certain genera have consistent overall behaviour (relative abundance) in a certain environment or not. Another rationale behind running the order-level neural network was that a lot of AAP genera, even with the most complete taxonomic database up to date, are still unclassified (e.g. genera of Rhizobiales order). Additionally, identification at species level was not possible with methodology used here, so there is an important taxonomic link missing between genus and ASVs, challenging the interpretation of this model. Hence, we focus on order- and genus- level data.

**Fig. 7:**
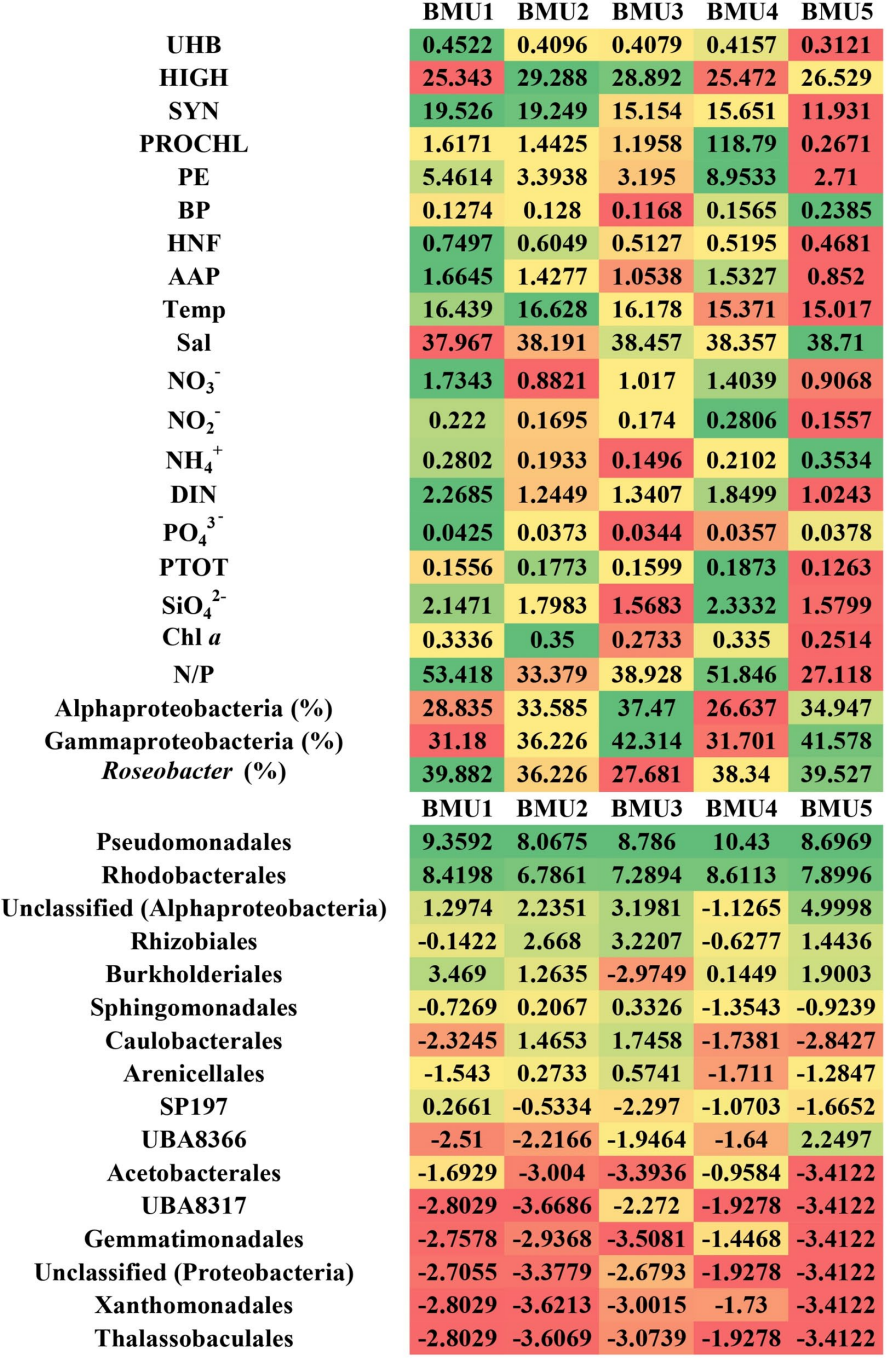
Neural gas analysis of *puf*M dataset agglomerated at the order level clustered into five distinctive BMUs according to their relative contribution to AAP community (expressed as average CLR-transformed values) and connected average values of biotic (UHB, HIGH, SYN, PROCHL, PE, BP, HNF, AAP, percentages of FISH-IR groups) and abiotic (Temp, Sal, NO_3_^−^, NO_2_^−^, NH_4_^+^, DIN, PO_4_^3−^, PTOT, SiO_4_^2−^, Chl *a,* N/P) variables of the environment. Colour gradient from red to green represents the lowest and highest average values respectively

AAPs agglomerated at the order level were clustered into five distinctive BMUs according to their relative contribution to community (Fig. 7, Additional file 7: Fig. S1, Additional file 8). Detailed description of each BMU unit is given in Additional file 7. BMUs generally described most nutrient-enriched (BMU1), warmest and shallowest (BMU2), lowest ammonia (BMU3), maximum nitrite (BMU4) and coldest, nutrient-depleted, deepest and most saline (BMU5) environment (Fig. 7). In all units, two orders were omnipresent and showed consistently high contribution regardless of environmental conditions: Pseudomonadales and Rhodobacterales. In contrast, orders Acetobacterales, UBA8317, Gemmatimonadales, Xanthomonadales and Thalassobaculales occurred transiently in higher, but mostly in very low values (Fig. 7).

**Fig. 8:**
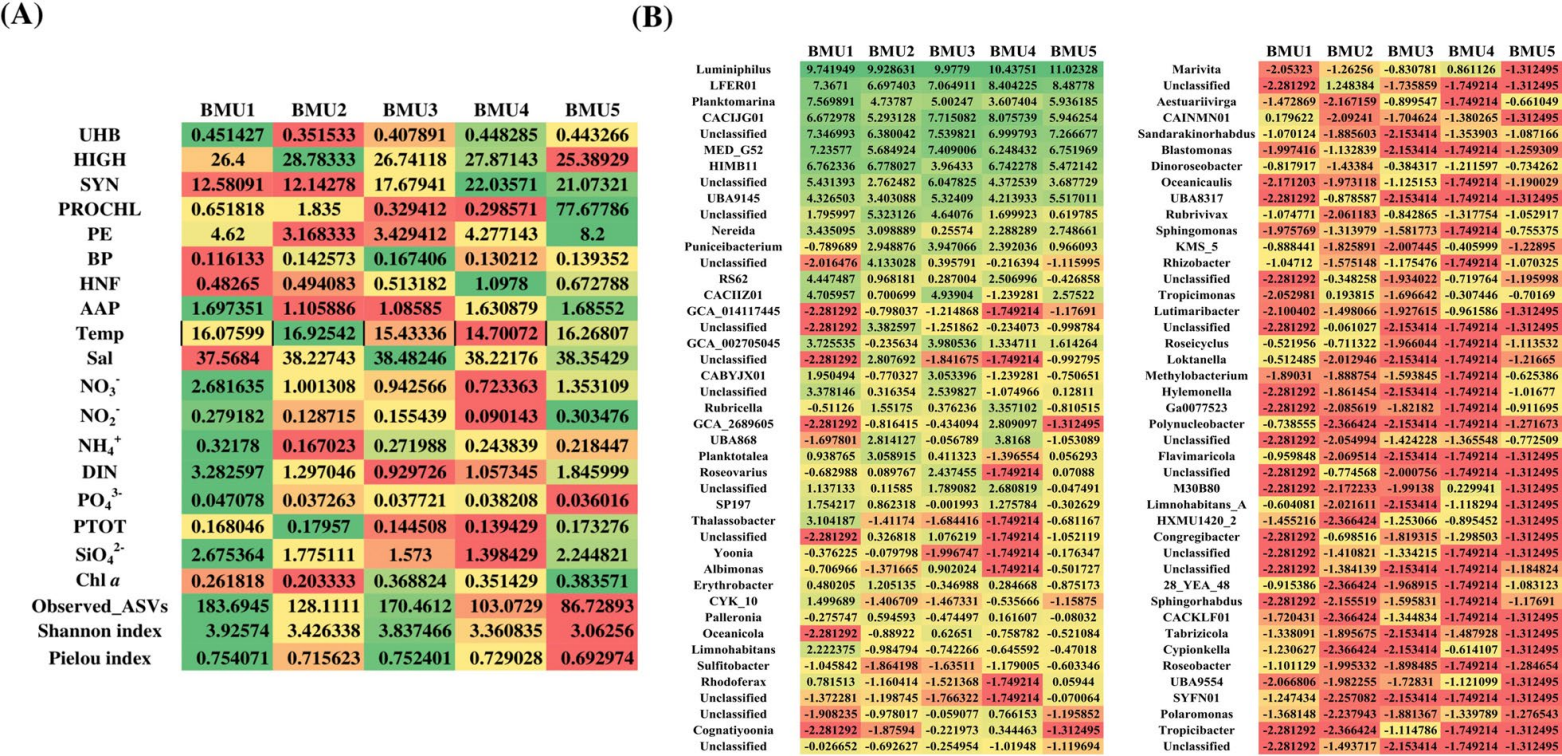
Neural gas analysis results of *puf*M dataset agglomerated at the genus-level and CLR-transformed, clustered into five BMUs. Biotic (UHB, HIGH, SYN, PROCHL, PE, BP, HNF, AAP, diversity metrics) and abiotic (Temp, Sal, NO_3_^−^, NO_2_^−^, NH_4_^+^, DIN, PO_4_^3−^, PTOT, SiO_4_^2−^, Chl *a,* N/P) variables of the environment are given in (**A**) as average value for each unit. Relative contribution of specific genera to AAP community (expressed as average CLR-transformed values) is shown in (**B**). Colour gradient from red to green represents the lowest and highest average values respectively

Based on the *puf*M dataset agglomerated at genus level, five distinct BMUs were also formed covering a total of 86 unique genera (Fig. 8, Additional file 7: Fig. S2 and Additional file 9). Detailed descriptions for each BMU are given in Additional file 7. Genera *Luminiphilus* (family Halieaceae, order Pseudomonadales, class Gammaproteobacteria), the uncultured *LFER01* as well as *Planktomarina* and *CACIJG01* (family Rhodobacteraceae, order Rhodobacterales, class Alphaproteobacteria) had almost the same high average values across all units. In nutrient-enriched and low-salinity habitat (BMU1), higher representation of *Nereida*, *CYK10*, *Thalassobacter,* and the Gammaproteobacteria *RS62*, *Rhodoferax* and *Limnohabitans* was noted, coupled with the highest abundance of AAPs, heterotrophic bacteria and picoeukaryotes (Fig. 8). Genera *Erythrobacter* (order Sphingomonadales), *Planktotalea* and *Palleronia* (Rhodobacteraceae) clustered with the lowest abundances of total heterotrophic bacteria, *Synechococcus*, picoeukaryotes and low abundances of AAPs as well as HNF, preferring the warmest habitat with low ammonia, Chl *a*, nitrate and nitrite (BMU2). The most saline yet diverse habitat scarce with nitrate and dissolved inorganic nitrogen, where AAP abundance was lowest, was dominated by the highest value of genera *Roseovarius*, *Puniceibacterium* and *CACIIZ01* (Rhodobacteraceae) and the gammaproteobacterial *CABYJX01* (Halieaceae, Pseudomonadales), indicating these genera do not appear to be controlled by a low-N environment (BMU3). The shallowest unit with a clear spatiotemporal pattern (almost all winter samples from transitional/open sea stations) with the lowest average temperature, scarce with nitrate, silicate, absolute abundances of *Prochlorococcus* but abundant with *Synechococcus*, HNF and AAPs were characterised by higher incidence of *CACIJG01*, *Rubricella, GCA2689605* (Alphaproteobacteria, family Hyphomicrobiaceae) and *UBA868* (Arenicellales), indicating their preference for colder environments (BMU4). Genera *Roseobacter* and *Sphingomonas*, rarely found in other units, preferred nutrient-rich habitat (BMU5), characterised by very high average salinity and temperature above 16 °C, rich in nitrite, silicate and Chl *a*, with maximum absolute abundances of *Prochlorococcus*, picoeukaryotes, high abundances of *Synechococcus* and AAPs, but with the lowest diversity (Fig. 8). In respect to order-level model, it was noticed that the most dominant genera inside a particular order (e.g. genus *Luminiphilus* of order Pseudomonadales and *LFER01* of order Rhodobacterales) masked rare ones, which did not follow general behaviour of the order. Examples include *Nereida* and *Puniciebacterium* of order Rhodobacterales which were inconsistently present in high values across units. Another example is rare genus *Rubrivivax* which expressed different behaviour from Burkholderiales order it belongs to (Figs. 7 and 8).

ASV-level NG model based on an entire ASV dataset (661 ASVs) also revealed five distinct units (BMUs). ASVs of the same genus exhibited different behaviours, but it was difficult to describe each specific variant of each genus. The most prominent variants of major genera that showed specific behaviour in certain BMU are shown in Additional file 7: Fig. S3. What can be observed is that in this model, we generally perceive more variation in respect to order- and genus-level models, *i.e*. certain ASVs appear only in some BMUs and are missing from others, which was masked at higher taxonomic levels.

## Discussion

Since AAPs represent a fascinating group of ubiquitous photoheterotrophs important for carbon cycling, they have been extensively studied in various environments in recent decades, especially in aquatic ones [10]. Previous research in the Adriatic Sea focused on their abundances estimated from Bchl *a* concentrations along a latitudinal transect [56], and on counts from coastal and estuarine waters along the eastern Adriatic coast in summer using infrared epifluorescence microscopy [22]. Subsequent studies based on one-year sampling along the trophic gradient described their spatial and vertical distribution with maximum abundances reported in late winter (April) [23]. A recent review article collected seven years of data from 34 sites to gain insights into their distribution and controlling environmental factors in the Adriatic Sea [24].

However, a comprehensive analysis of AAP community structure in the Adriatic Sea on a fine taxonomic scale has not been yet described. In this study, we present their community composition during one year in the central Adriatic from three stations along the trophic gradient using *puf*M metabarcoding and quantitative FISH-IR approach. In addition, neural gas algorithms were applied to agglomerated at order- and genus-level sequencing dataset (CLR-transformed after agglomeration) and ASV-level to reveal patterns of the AAP community in respect to specific physicochemical and biological variables.

In the study area, significant differences in seasonality were observed in terms of AAP abundances (maximum average value in spring: 2.136 ± 0.081 × 10^4^ cells mL^−1^, minimum in summer: 0.86 × 10^4^ cells mL^−1^), FISH-IR groups (*Roseobacter* prevalent in autumn, Alpha- and Gammaproteobacteria in summer) and *puf*M genus-agglomerated metabarcoding data. However, no clear spatial pattern emerged, as previously reported for the Mediterranean coastal lagoon [57]. The pronounced seasonality is consistent with the first long-term study based on *puf*M amplicon dataset from the northwestern Mediterranean, which was conducted over a decadal period and found that this community is highly seasonal, with certain taxa showing recurrent patterns [5].

### AAP abundances

As for the absolute abundances, the average values of 1.427 ± 0.754 × 10^4^ cells mL^−1^ correspond to those previously reported for the central Adriatic. However, the range of 0.30 × 10^4^ to 3.41 × 10^4^ cells mL^−1^ was narrower in our study, compared to studies that sampled Adriatic estuaries [22–24]. The average relative abundance of AAPs was 3.862% ± 2.266% of total prokaryotes (minimum of 0.548%, maximum of 10.258%), which is comparable to previous results from the Adriatic [22, 23] and the Mediterranean [4, 18, 57], as well as the Global Ocean Sampling Expedition [41]. As in the study by Ferrera et al. [18] where maximum abundances of AAPs were found in summer, a clear and significant seasonality was observed in our study. In contrast, we have detected the maximum average abundance in spring, the only season significantly different from the others in the pairwise comparison, and the minimum average abundances in summer. The maxima observed in April and June are comparable to a previous study in the Adriatic, where maximum AAP abundances were also reached in April at all stations, with increased phosphorus concentrations and lower N/P ratio as possible explanations [23]. Depth in the range from 0 to 100 m as well as dChlMax did not prove to be a significant environmental factor controlling AAP abundance in this study, suggesting that, contrary to previous results [23, 58], sea transparency of the central Adriatic was not a crucial factor affecting this community. Inconsistency in spatial and vertical distribution pattern in our study compared to previous research in Adriatic could be possibly explained by the trophic status of study area. Previous research covered more eutrophic coastal and estuarine area to the oligotrophic open sea [23], whilst in our study differences in trophic status were less enhanced and overall oligotrophic at all depths. Potential explanation for inconsistency in spatial distribution compared to previous results could be attributed to geographic proximity of stations studied in our research, while in previous ones larger study area was covered [22, 23].

### Quantitative estimates from FISH-IR

Quantitative estimates of specific bacterial groups resulting from fluorescence in situ hybridisation combined with catalysed reporter deposition (CARD-FISH) have been widely reported in numerous ecological studies for years [14, 27, 59–61]. Nevertheless, quantitative estimates of AAPs taxonomically assigned to specific clades or groups are rare [21, 29], especially for environmental samples. In our study, FISH-IR was applied to quantify the percentage of AAPs assigned to either the Gammaproteobacteria class, the Alphaproteobacteria class or the *Roseobacter* clade. Overall, the AAP community in the central Adriatic was quite heterogeneous according to these three probes, with the highest mean relative abundances of AAPs associated with *Roseobacter* clade, followed by gammaproteobacterial AAPs and alphaproteobacterial ones. A distinct seasonal pattern emerged, with AAPs assigned to *Roseobacter* clade generally less abundant in warmer months and more prevalent in autumn, while the patterns of general Alphaproteobacteria and Gammaproteobacteria were inverse to those of *Roseobacter*. The proposed term *Roseobacter* clade stands for a group of marine Rhodobacteraceae (members of the Rhodobacterales order that share 89% identity of 16S rRNA gene sequences), which are known to be ubiquitous in seawater and crucial for biogeochemical cycling of various elements [62]. Most of the cultured members have quite large genomes, high GC content (~ 60%) and a wide diversity of metabolic capabilities. Both free-living and/or associated with phytoplankton and microalgae, *Roseobacter*-like bacteria could account for up to 30% of bacterial communities in marine environments [27, 62, 63]. The lower contribution of AAPs assigned to this clade observed in summer contrasts with previous findings where the *Roseobacter* clade (represented as % of total bacteria, determined via CARD-FISH) had the highest average relative abundances in summer [27, 64]. However, the capability for aerobic anoxygenic phototrophy, recruited via horizontal gene transfer, is considered to be present in at least 14 phylogenetically distinct strains of roseobacters, but not in all *Roseobacter*-clade members [62, 65]. Therefore, we hypothesize that the seasonal behaviour of AAPs that belong to *Roseobacter* clade may potentially differ from other *Roseobacter*-clade members due to AAPs’ advantage of photoheterotrophy (*i.e.* heterotrophic energy acquisition through oxidation of organic matter coupled with light utilization) [62]. Nevertheless, FISH-IR data in conjunction with the NG order-level model revealed that AAP roseobacters prefer a warm, less saline and nutrient-enriched environment (order-level BMU1, without distinct seasonality observed), associated with the highest absolute abundances of AAPs, suggesting that *Roseobacter*-like bacteria dominate the AAP community in the described environment. Another pattern significant for roseobacters was their preference for ammonia regardless of ambient temperature, with their lowest counts in BMU3 coinciding with the lowest ammonia concentrations and vice versa in an ammonia-enriched habitat (BMU5). This is to be expected as many organisms in the *Roseobacter* clade can utilise nitrogen exclusively in a reduced form, as they do not possess genes encoding enzymes for nitrate and nitrite reduction, but gene clusters encoding enzymes for the uptake of ammonium, amino acids, spermidine and urea [66]. The observed seasonal dynamics of Alphaproteobacteria AAPs, characterised by the highest average relative abundances in summer and the lowest ones in autumn, is in contrast to previously reported seasonal behaviour of total Alphaproteobacteria estimated with CARD-FISH [27, 64, 67]. These results contradict the assumption that Alphaproteobacteria are generally good competitors in oligotrophic environments (predominant in winter months), suggesting that the behaviour of Alphaproteobacteria AAPs may differ from general bacterial community patterns, preferring eutrophic habitats despite their phototrophic capabilities [7, 10, 68]. In contrast, the peak abundances of gammaproteobacterial AAPs in summer are comparable to previous findings about general Gammaproteobacteria, indicating their preference for higher nutrient concentrations (mainly total nitrogen), assimilation of organic carbon sources and generally higher temperatures [27, 68].

When discussing these results, it is crucial to bear in mind the substantial limitations of FISH-IR method as well as the coverage and specificity of oligonucleotide probes. As mentioned in Results, percentage of AAP cells hybridized with the ROS537 probe was generally higher than the number of cells detected with the ALF968 probe, sometimes leading to a total sum of relative abundances greater than 100%. Similar results were also observed in other studies for same probes, but for CARD-FISH results [27, 69, 70]. Riou et al*.* [71] re-evaluated the in silico specificity of eleven bacterial and eukaryotic probes regularly used to enumerate marine microbes via CARD-FISH and found that both the Ros537 and Roseo536R probes identified 91% of the alphaproteobacterial *Roseobacter* clade, but also targeted 2.9% of sequences classified as non-*Roseobacter* (other members of the Rhodobacterales order). Similar result was observed in our study when in silico evaluating ROS537 probe, with coverage of 92.9% and specificity of 99.3%. In addition, previous studies reported false-positive hits and misclassification of marine Gammaproteobacteria with the Gam42a probe and of Alphaproteobacteria with the ALF968 probe, with group coverage of 76% and 81%, respectively [72]. In our study, ALF968 probe showed higher in silico coverage (96.3%) of Rhodobacterales order than ROS537 probe, but with lower specificity (~ 93%). Hence, we speculate that superior complementary base pairing close to 100% of ROS537 probe, especially for genera *Nereida*, *Planktomarina* and *Puniceibacterium* detected in higher relative abundances with *puf*M metabarcoding, could explain higher relative abundances observed with ROS537 than ALF968. This may indicate an urgent need for FISH probes redesign, especially ALF968. Furthermore, almost all Alphaproteobacteria (96%) detected in this study belong to the *Roseobacter* clade (*i.e.* Rhodobacterales order, Rhodobacteraceae family) as revealed by metabarcoding results. Considering decreased specificity of the ALF968 probe, this might explain why numbers fall below that of ROS537 probe. Other major disadvantages of FISH, besides the coverage and affinity of the probes for complementary nucleic acids, are low and variable number of ribosomes (targets) in certain bacterial cells, the lack of cells’ permeabilization and discrepancies in the binding of fully complementary oligonucleotides, discussed in detail elsewhere [72]. Another substantial limitation of this method that may have affected the results was the rapid fading of Bchl *a* autofluorescence when infrared and probe signals were simultaneously detected, which often required rapid and repeated manipulation when images were acquired. Nevertheless, positive correlation between FISH-IR and *puf*M pseudoabundances for corresponding taxonomic levels indicate that FISH-IR data image well relative patterns observed in AAP community, while Gam42a and ROS537 probes contribute solid quantitative estimates of these groups. This becomes important when considering inherent primer bias in metabarcoding, especially in respect to Gammaproteobacteria (see further below) where FISH-IR data provide alternative estimate and added value to the dataset (35.3% FISH-IR Gam42 vs. 75.9% *puf*M metabarcoding). These findings pave the way for protocol optimizations and new probe designs targeting AAPs.

### Community structure from *puf*M gene metabarcoding

Although we have obtained taxonomic resolution at the genera level, for the purpose of comparison to previously published studies, taxonomic phylogroups proposed by Yutin et al. [41] will be referred to if they have a taxonomically described representative in our dataset. The community composition determined by *puf*M gene sequencing is consistent with the results of Auladell et al. [5] from the northwestern Mediterranean region mentioned earlier, where Gammaproteobacteria belonging to the NOR5/OM60 group (phylogroup K; Pseudomonadales) dominated the AAP composition over a decade with a mean relative abundance of 83.8%, while their mean relative contribution in our study was 75.9%. Interestingly, the similar decrease in the contribution of Gammaproteobacteria as in Auladell et al. [5] for months of February and March (59.6% and 52% on average, respectively) was also observed in our study, with a relative respective contribution of < 40% and 50%. In these months, we detected an increased incidence of Alphaproteobacteria, specifically order Rhodobacterales, in February for ST101 and in March for CJ007. Order Rhodobacterales in the study area was associated with phylogroups E, F and G. Occasional peaks of certain AAPs with relative contributions > 1%, as reported in other studies, were also present in our survey. For instance, Sphingomonadales peaked in April, Caulobacterales in August, and the genus *Planktomarina* (phylogroup E) in February and November [5, 7]. However, a long-term survey is required to assess whether these occurrences are repeatable.

At the genus level, community composition in our study area was dominated by Gammaproteobacterium *Luminiphilus* (phylogroup K, OM60/NOR5 clade; order Pseudomonadales, family Halieaceae), a mesophilic and moderately halophilic photoheterotroph common in seawater and surface layers of littoral marine sediments [73]. Besides *Luminiphilus*, taxon with the highest relative contribution to AAP community composition was *LFER01*, belonging to Rhodobacterales order (phylogroup G, family Rhodobacteraceae). Of interest is the report of two Gemmatimonadota ASVs at transition station CJ007, the first report of Gemmatimonadota AAPs in marine habitat, although they were present in a very low relative abundance. This indicates that amplicon sequencing provides a suitable tool for detection of very low abundant (0.1–10%) taxa, compared to metagenomics [19]. Gemmatimonadota is a poorly studied bacterial phylum, with only six cultivated representatives reported to date, whose photoheterotrophic and heterotrophic members are commonly found in soil, euphotic zones of freshwaters, sewage treatment plants and sediments, however their photoheterotrophic members were not yet detected in marine habitats [74, 75]. Two ASVs observed in our study were assigned to unclassified genera of the family Gemmatimonadaceae. Currently, only two cultivated representatives, *Gemmatimonas phototrophica* and *Gemmatimonas groenlandica*, are known to possess the capacity for anoxygenic photosynthesis. However, metagenome-assembled genome analyses have shown that anoxygenic photosynthesis is also present in uncultivated lineages of Gemmatimonadota [74, 76]. We hypothesise that the Gemmatimonadota detected in our study may originate from the river basin of the Adriatic karst rivers, namely Jadro and Cetina, or are passively transported from the sediment.

As previously confirmed by different methodological approaches such as metagenomics [5, 77], amplicon sequencing [5, 7] and microscopy [18], our results also indicate an inverse relationship between AAP abundance and diversity metrics in summer. The highest AAP diversity in relation to the Shannon index and Pielou’s evenness was found when AAP abundance was the lowest in August. This contrasts with previous results from a long-term study by Auladell et al. [5] (NW Mediterranean), where diversity was highest (when abundance was lowest) in winter. In our study, no clear inverse relationship was found between AAP abundance and diversity measures in other seasons.

Although amplicon sequencing is cost-effective compared to metagenomics, primer biases due to high variability in protein-coding gene sequences, such as in the *puf*M gene, remain a major drawback of this approach [19]. A recent article by Gazulla et al. [19] compared metagenomic and metabarcoding approaches using existing and newly designed primers (pufMF/pufM WAW, UniF/UniR and pufMF_Y/pufM WAW) and showed that all primer pairs are biased towards Gammaproteobacteria and even some members of Alphaproteobacteria. In our study, we used UniF/WAW primer pair combination and obtained successful PCR amplification on marine samples, unlike Gazulla et al. [19] (see Additional file 1: Table S2). However, it is reasonable to expect these primers also possibly overestimate the Gammaproteobacterial AAPs (Pseudomonadales, phylogroup K) to the detriment of uncultured representatives and results should be interpreted with caution. As mentioned previously, *puf*M metabarcoding estimates relative contribution of Gammaproteobacteria to 75.9% while FISH-IR Gam42 to 35.3%. Moreover, PCR reaction optimisation was lengthy and tedious, possibly due to the very low GC content of UniF, the low melting temperature and ten degenerate nucleotides in the primer sequence. Instead, the newly designed forward primer pufMF_Y in combination with the reverse primer WAW and the universal UniF/UniR primer pair are currently proposed to obtain a less biased taxonomic representation of marine AAP [19].

### Environmental variables affecting AAP community composition

Neural gas models linking sequencing data to biotic/abiotic environmental factors have already been successfully applied to 16S rRNA gene datasets [25, 27]. Here, they were performed at order-, genus- and ASV-levels of the *puf*M dataset to extract characteristic patterns that could potentially elucidate still unresolved ecology of AAPs. In general, the Adriatic Sea is an oligotrophic environment, scarce in nitrogen and phosphorus, with low productivity and elevated average salinity [78, 79]. In order- and genus-NG models, the maximum abundance of AAPs was detected in more productive environments (BMU1 in both and BMU5 in the genus-NG model), namely those with the highest concentrations of soluble reactive phosphorus, nitrate and dissolved inorganic nitrogen, consistently coupled with the highest abundance of total heterotrophic bacteria [10]. The gammaproteobacterial order Burkholderiales (phylogroup I), represented by the genera *Rhodoferax* and *Limnohabitans*, as well as the alphaproteobacterial lineage *SP197* and genera belonging to order Rhodobacterales, family Rhodobacteraceae *Planktomarina* (phylogroup E), *Nereida* (phylogroup G), *CYK10* and *Thalassobacter* (phylogroup E) were present in increased relative abundances in nutrient-enriched environments with the highest AAP diversity. Conversely, the abundance of AAPs was lowest in nutrient-poor habitats with low nitrogen and phosphorus, according to order- and genus-model. Another feature evident in aforementioned models is the inverse relationship between AAP abundance and salinity as a potentially important controlling factor: the lowest abundance was found in the most saline habitats and vice versa. However, in contrast to previous research from saline lakes of the Tibetan Plateau, neither clear nor inverse relationship between diversity measures and salinity was found in our study [80]. The observed number of ASVs and Shannon index reached a maximum in habitats with the lowest salinity, while a similarly high number of ASVs, Shannon index and highest Pielou’s evenness were found in habitats with the highest salinity (BMU3), suggesting that AAP diversity is not necessarily directly impacted by salinity. Furthermore, the high AAP diversity in different habitats, both nutrient-rich (BMU1) and nutrient-poor one (BMU3), was independent of temperature or salinity in the studied area, again indicating a different behaviour of AAPs compared to the general bacterial population. Previous results focusing on bacteria and archaea in the Adriatic Sea pointed to the "*plankton paradox*": archaeal and bacterial diversity is lowest in the environment with the highest abundance of picoplankton members, bacterial production and chlorophyll concentration, while, on the contrary, the highest bacterial diversity was measured in deep and saline environments abundant with nitrates, nitrites and soluble reactive phosphorus [25, 27]. Nevertheless, a high average salinity (38.35) combined with a temperature greater than 16 °C associated with the highest concentrations of nitrites, silicates and Chl *a* negatively affected AAP diversity (BMU 5). In contrast to previous studies [5, 24], there was neither a clear nor a linear relationship between Chl *a* (coupled with cyanobacterial abundance peaks), and the abundance of AAPs in our order- and genus-level models.

The alphaproteobacterial orders Rhizobiales (phylogroup J), Sphingomonadales, Caulobacterales and Arenicellales occurred simultaneously in higher incidence in habitats scarce with nitrate/ammonia and temperatures above 16 °C (BMUs 2 and 3). Conversely, they had low values in nitrogen-enriched environments (BMUs 1 and 4), indicating an inverse relationship between their relative abundances and nitrogen compounds and possibly their preference for low-nitrogen habitats. Caulobacterales, Rhizobiales and Sphingomonadales also co-occurred in a recent study conducted in freshwater lakes, possibly indicating a preference of these orders for the same environmental conditions, with their maxima observed in spring [7]. Recently, the contribution of AAP photoheterotrophic activity to carbon fluxes in a freshwater lake was quantified and associated with higher relative abundance of Caulobacterales and Sphingomonadales, possibly implying an important role of these orders in aquatic habitats [17]. Interestingly, the uncultured alphaproteobacterial lineage UBA8366 and an unclassified order of Alphaproteobacteria showed the highest incidence only in the coldest, scarcest, and deepest environment (order-level BMU5) with an average depth of ~ 60 m and maximum average salinity (38.71), which coincided with the lowest absolute abundances of AAPs, total heterotrophic bacteria, *Synechococcus*, *Prochlorococcus*, picoeukaryotes, HNF and diversity metrics. This result is consistent with a previous study that found a dominance of Alphaproteobacteria in deeper and saltier seawater [27]. A recent article dealing with the comparative genomics of Alphaproteobacteria MAGs from the Arctic Ocean also showed that the uncultured UBA8366 lineage is involved in the degradation of aromatic compounds, especially humic substances of terrestrial origin [81].

To our knowledge, there is no previous literature to compare data obtained at genus- and ASV-level analysis in respect to sea-water environmental conditions. In addition to the eurivalent genera *Luminiphilus* (phylogroup K, OM60/NOR5 clade; order Pseudomonadales, family Halieaceae) and the uncultured *LFER01* (phylogroup G, order Rhodobacterales, family Rhodobacteraceae), which were prevalent in all five distinct environments (BMU1-5 at the genus level), alphaproteobacterial genera *Planktomarina* (phylogroup E), *Nereida* (phylogroup G), *CYK10*, *Thalassobacter* (phylogroup E) from family Rhodobacteraceae and the Gammaproteobacteria *RS62*, *Rhodoferax* and *Limnohabitans* (phylogroup I, family Burkholderiaceae) appeared in highest values in nutrient-enriched habitat (genus–level BMU1), coupled with the highest abundance of AAPs, heterotrophic bacteria and picoeukaryotes. These genera preferred low-salinity environments enriched in nitrates, ammonia, dissolved inorganic nitrogen, phosphorus and silicates. As already mentioned, this is to be expected as *Roseobacter* group members, such as genus *Planktomarina*, utilise nitrogen only in reduced form as they lack the metabolic pathways for nitrate and nitrite reduction and rely on the uptake of ammonium, amino acids, spermidine and urea [66]. Regarding ASV-level neural network, it was observed that different variants of the same genus behaved differently when clustered in specific environment. However, working at the lowest taxonomic resolution possible did not result in easily interpretable/straightforward model which could be compared to previous literature and order- and genus-level BMUs. We contribute this to the fact that we are missing species level taxonomic identification and interpretation was focused on higher taxonomic rank models instead.

## Conclusion

Overall, significant seasonality was observed regarding AAP abundances (maximum average values in spring and minimum in summer), FISH-IR data (*Roseobacter* clade prevalent in autumn, Alpha- and Gammaproteobacteria in summer) and *puf*M sequencing dataset (highest diversity metrics in summer). Community composition in Adriatic Sea based on *puf*M metabarcoding was dominated by Gammaproteobacteria belonging to the NOR5/OM60 clade, namely the genus *Luminiphilus*, while numerous other genera were present in low relative abundances < 1%. An inverse relationship between AAP abundance and diversity metrics was observed in summer. Neural gas models revealed potentially important controlling variables of AAP abundance, community structure, and diversity measures. The high AAP diversity was independent of temperature or salinity in various trophic environments, indicating a different behaviour of AAPs compared to the general bacterial population. We emphasize there is need for redesign of FISH-IR Alphaproteobacterial hybridization probe, however, positive correlation between FISH-IR and *puf*M metabarcoding datasets indicate that FISH-IR provides solid quantitative estimates of relative abundances of Gammaprotebacteria (Gam42a probe) and *Roseobacter* clade (ROS537 probe). This represents an added value of combining qualitative and quantitative approaches, especially when considering inherent primer bias towards Gammaproteobacteria in *puf*M metabarcoding.

### Supplementary Information

**Additional file 1**. **Table S1**. Description of samples analysed in this study ordered by month (date of collection, W-Winter, Sp-Spring, S-Summer, A-Autumn).; **Table S2**. Number of reads per sample that passed through dada2 pipeline; input: number of raw reads, filtered: filtered out low quality sequences and tails, denoisedF and denoisedR: denoised sequences after the core sample inference algorithm of forward and reverse reads respectively, merged: merged sequenceswhich are output if the forward and reverse reads overlap by at least 12 bases and are identical to each other in the overlap region, nonchim: sequences after chimera removal [1]. Samples with an unacceptably low final number of reads per sample (N<2000) were excluded from further analysis.; **Figure S1**. Rarefaction curve exhibiting sufficient sequencing depth (Sample Size) for AAP diversity metrics estimates. Rarefying to the smallest library size was performed multiple times (function rarefy_even_depth from phyloseq v1.32.0 R package [2], rarefying threshold =2000, N(times) =100)., **Figure S2**. Draftsman plots used to estimate correlation of abiotic environmental variables, made in PRIMER7 [3]. Variables are: temperature-Temp, salinity-Sal, nitrates-NO3-, nitrites-NO2-, ammonium ion-NH4, dissolved inorganic nitrogen-DIN, total nitrogen-NTOT, soluble reactive phosphorus-SRP, total phosphorus-PTOT, silicate-SiO4., **Table S3**. Permutational multivariate analysis of variance (PERMANOVA) on Euclidean distance of square-root transformed AAPs’ absolute (A) and relative (B) abundance dataset. Factors: Se-Season (fixed), Re-Region (fixed), La- Layer (nested in Region, L1 (0-30m), L2 (30-50m), L3 (50-75m), L4 (75-100m)). Pairwise comparisons for significant seasonality (W-Winter, Sp-Spring, S-Summer, A-Autumn) are given in the right part of the table. PERMANOVA was performed in PRIMER7 with 9999 permutations, unrestricted permutation of raw data, sums of squares type: Type II (conditional) [3]., **Table S4**. Permutational multivariate analysis of variance (PERMANOVA) on Aitchison distance of centered-log ratio transformed pufM sequencing dataset agglomerated on genus-level. Factors: Se- Season (fixed), Re-Region (fixed), La- Layer (nested in Region, L1 (0-30m), L2 (30-50m), L3 (50-75m), L4 (75-100m)). Pairwise comparisons for significant seasonality (W-Winter, Sp-Spring, S-Summer, AAutumn) are given in the right part of the table. PERMANOVA was performed in PRIMER7 with 9999 permutations, unrestricted permutation of raw data, sums of squares type: Type II (conditional) [3], **Figure S3**. AAP community composition obtained via pufM metabarcoding in the study area shown at the class level per station (ST101, CJ007, CJ009), month and depth., **Table S5**. Alpha diversity metrics based on rarefied pufM dataset (Observed number of ASVs, Shannon and Pielou’s index) given as average values with standard deviations (SD) calculated per season (vegan v2.5.7 R package [4]).

**Additional file 2**. Detailed description of Infrared epifluorescence microscopy and fluorescence in situ hybridisation (FISH-IR). **Table S1**. Permutational multivariate analysis of variance (PERMANOVA) on Bray-Curtis similarity for square-root transformed FISH-IR relative abundance dataset. Factors: Se- Season (fixed), Re-Region (fixed), La- Layer (nested in Region, L1 (0-30m), L2 (30-50m), L3 (50-75m), L4 (75-100m)). Pairwise comparisons for significant seasonality (W-Winter, Sp-Spring, S-Summer, A-Autumn) are given in the right part of the table. PERMANOVA was performed in PRIMER7 with 9999 permutations, Unrestricted permutation of raw data, sums of squares type: Type II (conditional) [5]., **Figure S1**. Two-dimensional nonmetric multidimensional scaling (NMDS) ordination plot of FISH-IR square root transformed dataset (relative abundances of AAPs assigned to Alphaproteobacteria, Gammaproteobacteria and Roseobacter clade) on a seasonal scale (W-Winter, Sp-Spring, S-Summer, A-Autumn)., **Figure S2**. Spatio-temporal distribution of FISH-IR groups (probes for Alphaproteobacteria, Gammaproteobacteria, Roseobacter) given as relative abundances of AAPs (n=90 samples), shown per station (ST101, CJ007 and CJ009), month, and depth., **Figure S3**. Seasonal distribution (W-winter, Sp-Spring, S-Summer, A-Autumn) of FISH-IR groups (Alphaproteobacteria, Gammaproteobacteria, Roseobacter) given as average relative abundances of AAPs.

**Additional file 3**. Sample data, untransformed final ASV matrix, taxonomy and reference pufM sequences (refseq).

**Additional file 4**. Correlation between metabarcoding and FISH-IR data; Spearman correlation coefficients between pseudoabundances from pufM sequencing (i.e. relative abundance of Gammaproteobacteria class × AAP absolute number/100) and FISH-IR counts.

**Additional file 5**. In silico coverage and specificity of ALF968 and ROS537 probes.

**Additional file 6**. Spatio-temporal distribution of Luminiphilus ASVs. **Figure 1**. Genus Luminiphilus composition via pufM metabarcoding at ST101 station shown per month and depth. Category “Other” represents ASVs with relative abundances below 5%., **Figure 2**. Genus Luminiphilus composition via pufM metabarcoding at CJ007 station shown per month and depth. Category “Other” represents ASVs with relative abundances below 5%., **Figure 3**. Genus Luminiphilus composition via pufM metabarcoding at CJ009 station shown per month and depth. Category “Other” represents ASVs with relative abundances below 5%.

**Additional file 7**. Detailed description of neural gas analysis results. **Figure S1**. Heatmap of order-agglomerated and CLR-transformed pufM dataset resulting in five distinct BMUs. Samples are shown for each unit., **Figure S2**. Genus-agglomerated and CLR-transformed pufM dataset resulting in five distinct BMUs. Samples are shown for each BMU., **Figure S3**. Neural gas analysis results of pufM dataset at clr-transformed ASV-level, clustered into five BMUs. Biotic (UHB, HIGH, SYN, PROCHL, PE, BP, HNF, AAP) and abiotic (Temp, Sal, NO3-, NO2-, NH4+, DIN, NTOT, PO43-, PTOT, SiO42-, Chl a) variables of each environment are given in (A) as average value for each unit. Colour gradient from red to green represents the lowest and highest average values respectively. Relative contribution expressed as CLR-transformed value of specific ASV is shown in (B). ASVs are ordered by genus, with most dominant genera ordered alphabetically.

**Additional file 8**. Neural gas analysis at the order level: sample data with resulting BMUs for each sample, ASV matrices and taxonomy table.

**Additional file 9**. Neural gas analysis at the genus level: sample data with resulting BMUs for each sample, ASV matrices and taxonomy table.

## Data Availability

The datasets supporting the results of this article are included within the article and its additional files. The raw sequencing dataset supporting the conclusions of this article is available in the National Center for Biotechnology Information (NCBI) Sequence Read Archive (SRA) as a part of BioProject PRJNA912619 under accession numbers SAMN37596707-SAMN37596796.

## Abbreviations

AAPs: Aerobic anoxygenic phototrophs
ASV: Amplicon sequence variant
BMU: Best match unit
BChl a: Bacteriochlorophyll-*a*
BP: Bacterial production
Chl *a*: Chlorophyll *a*
CLR: Centered log-ratio
DAPI: 4’,6-Diamidino-2-phenylindole
dChlMax: Deep chlorophyll maximum
DIN: Dissolved inorganic nitrogen,
FISH: Fluorescence in situ hybridisation
FISH-IR: Infrared epifluorescence microscopy and fluorescence in situ hybridisation
HNA: High nucleic acid
HNF: Heterotrophic nanoflagellates
LNA: Low nucleic acid
NG: Neural gas
NH_4_^+^: Ammonium ion
NO_2_^−^: Nitrites
NO_3_^−^: Nitrates
NTOT: Total nitrogen
PE: Picoeukaryotes
PROCHL: *Prochlorococcus*
PTOT: Total phosphorus
Sal: Salinity
SiO_4_^2^^−^: Silicate
SRP: Soluble reactive phosphorus
SYN: *Synechococcus*
Temp: Temperature
UHB: Total heterotrophic bacteria
