# Supplementary material for: Ecology of aerobic anoxygenic phototrophs on a fine-scale taxonomic resolution in Adriatic Sea unravelled by unsupervised neural network
Source: Environ Microbiome. 2024 Apr 29;19:28. doi: 10.1186/s40793-024-00573-6 (PMC11059731; doi:10.1186/s40793-024-00573-6)
Supplement: Supplementary file 1 — Additional file 1. Table S1. Description of samples analysed in this study ordered by month (date of collection, W-Winter, Sp-Spring, S-Summer, A-Autumn).; Table S2. Number of reads per sample that passed through dada2 pipeline; input: number of raw reads, filtered: filtered out low quality sequences and tails, denoisedF and denoisedR: denoised sequences after the core sample inference algorithm of forward and reverse reads respectively, merged: merged sequenceswhich are output if the forward and reverse reads overlap by at least 12 bases and are identical to each other in the overlap region, nonchim: sequences after chimera removal [1]. Samples with an unacceptably low final number of reads per sample (N<2000) were excluded from further analysis.; Figure S1. Rarefaction curve exhibiting sufficient sequencing depth (Sample Size) for AAP diversity metrics estimates. Rarefying to the smallest library size was performed multiple times (function rarefy_even_depth from phyloseq v1.32.0 R package [2], rarefying threshold =2000, N(times) =100)., Figure S2. Draftsman plots used to estimate correlation of abiotic environmental variables, made in PRIMER7 [3]. Variables are: temperature-Temp, salinity-Sal, nitrates-NO3-, nitrites-NO2-, ammonium ion-NH4, dissolved inorganic nitrogen-DIN, total nitrogen-NTOT, soluble reactive phosphorus-SRP, total phosphorus-PTOT, silicate-SiO4., Table S3. Permutational multivariate analysis of variance (PERMANOVA) on Euclidean distance of square-root transformed AAPs’ absolute (A) and relative (B) abundance dataset. Factors: Se-Season (fixed), Re-Region (fixed), La- Layer (nested in Region, L1 (0-30m), L2 (30-50m), L3 (50-75m), L4 (75-100m)). Pairwise comparisons for significant seasonality (W-Winter, Sp-Spring, S-Summer, A-Autumn) are given in the right part of the table. PERMANOVA was performed in PRIMER7 with 9999 permutations, unrestricted permutation of raw data, sums of squares type: Type II (conditional) [3]., Table S4. Permutational mul [file 40793_2024_573_MOESM1_ESM.docx]

**Additional file 1**

**Ecology of Aerobic Anoxygenic Phototrophs on a fine-scale taxonomic resolution in Adriatic Sea unravelled by unsupervised neural network**

Iva Stojan^1,2^, Danijela Šantić^1^*, Cristian Villena-Alemany^3,4^, Željka Trumbić^5^, Frano Matić^5^, Ana Vrdoljak Tomaš^1^, Ivana Lepen Pleić^1^, Kasia Piwosz^6^, Grozdan Kušpilić^1^, Živana Ninčević Gladan ^1^, Stefanija Šestanović^1^, Mladen Šolić^1^

^1^ Institute of Oceanography and Fisheries, Šetalište Ivana Meštrovića 63, Split, Croatia

^2^ Doctoral Study of Biophysics, Faculty of Science, University of Split, Ruđera Boškovića 37, Split, Croatia

^3^ Laboratory of Anoxygenic Phototrophs, Institute of Microbiology, Czech Academy of Sciences, 37981 Třeboň, Czechia

^4^ Department of Ecosystem Biology, Faculty of Science, University of South Bohemia, České Budějovice, Czechia

^5^ University Department of Marine Studies, University of Split, Ruđera Boškovića 37, Split, Croatia

^6^ Department of Fisheries, Oceanography and Marine Ecology, National Marine Fisheries Research Institute, Gdynia, Poland

*Danijela Šantić, Institute of Oceanography and Fisheries, Šetalište Ivana Meštrovića 63, Split, Croatia, e-mail: [segvic@izor.hr](mailto:segvic@izor.hr)

**Number of pages: 12**

**Number of tables: 5**

**Number of figures: 3**

**Table S1.** Description of samples analysed in this study ordered by month (date of collection, W-Winter, Sp-Spring, S-Summer, A-Autumn).

| **Sample_ID** | **Sample abbreviation (station, depth, month)** | **Station** | **Depth [m]** | **Month** | **Season** |
| --- | --- | --- | --- | --- | --- |
| **1puf** | 101-0-2 | ST101 | 0 | 2 | W |
| **2puf** | 101-35-2 | ST101 | 35 | 2 | W |
| **3puf** | 007-0-2 | CJ007 | 0 | 2 | W |
| **4puf** | 007-30-2 | CJ007 | 30 | 2 | W |
| **5puf** | 007-50-2 | CJ007 | 50 | 2 | W |
| **6puf** | 009-0-2 | CJ009 | 0 | 2 | W |
| **7puf** | 009-28.5-2 | CJ009 | 28.5 | 2 | W |
| **8puf** | 009-50-2 | CJ009 | 50 | 2 | W |
| **9puf** | 009-75-2 | CJ009 | 75 | 2 | W |
| **10puf** | 009-100-2 | CJ009 | 100 | 2 | W |
| **31puf** | 101-0-3 | ST101 | 0 | 3 | W |
| **32puf** | 101-35-3 | ST101 | 35 | 3 | W |
| **33puf** | 007-0-3 | CJ007 | 0 | 3 | W |
| **34puf** | 007-30-3 | CJ007 | 30 | 3 | W |
| **35puf** | 007-50-3 | CJ007 | 50 | 3 | W |
| **36puf** | 009-0-3 | CJ009 | 0 | 3 | W |
| **37puf** | 009-37.5-3 | CJ009 | 37.5 | 3 | W |
| **38puf** | 009-50-3 | CJ009 | 50 | 3 | W |
| **39puf** | 009-75-3 | CJ009 | 75 | 3 | W |
| **40puf** | 009-100-3 | CJ009 | 100 | 3 | W |
| **71puf** | 101-0-4 | ST101 | 0 | 4 | W |
| **72puf** | 101-35-4 | ST101 | 35 | 4 | W |
| **73puf** | 007-0-4 | CJ007 | 0 | 4 | W |
| **74puf** | 007-30-4 | CJ007 | 30 | 4 | W |
| **75puf** | 007-50-4 | CJ007 | 50 | 4 | W |
| **76puf** | 009-0-4 | CJ009 | 0 | 4 | W |
| **77puf** | 009-25-4 | CJ009 | 25 | 4 | W |
| **78puf** | 009-50-4 | CJ009 | 50 | 4 | W |
| **79puf** | 009-75-4 | CJ009 | 75 | 4 | W |
| **80puf** | 009-100-4 | CJ009 | 100 | 4 | W |
| **11puf** | 101-0-5 | ST101 | 0 | 5 | Sp |
| **12puf** | 101-35-5 | ST101 | 35 | 5 | Sp |
| **13puf** | 007-0-5 | CJ007 | 0 | 5 | Sp |
| **14puf** | 007-30-5 | CJ007 | 30 | 5 | Sp |
| **15puf** | 007-50-5 | CJ007 | 50 | 5 | Sp |
| **16puf** | 009-0-5 | CJ009 | 0 | 5 | Sp |
| **17puf** | 009-30-5 | CJ009 | 30 | 5 | Sp |
| **18puf** | 009-50-5 | CJ009 | 50 | 5 | Sp |
| **19puf** | 009-68-5 | CJ009 | 68 | 5 | Sp |
| **20puf** | 009-100-5 | CJ009 | 100 | 5 | Sp |
| **41puf** | 101-0-6 | ST101 | 0 | 6 | Sp |
| **42puf** | 101-35-6 | ST101 | 35 | 6 | Sp |
| **43puf** | 007-0-6 | CJ007 | 0 | 6 | Sp |
| **44puf** | 007-30-6 | CJ007 | 30 | 6 | Sp |
| **45puf** | 007-50-6 | CJ007 | 50 | 6 | Sp |
| **46puf** | 009-0-6 | CJ009 | 0 | 6 | Sp |
| **47puf** | 009-30-6 | CJ009 | 30 | 6 | Sp |
| **48puf** | 009-60-6 | CJ009 | 60 | 6 | Sp |
| **49puf** | 009-75-6 | CJ009 | 75 | 6 | Sp |
| **50puf** | 009-100-6 | CJ009 | 100 | 6 | Sp |
| **61puf** | 101-0-8 | ST101 | 0 | 8 | S |
| **62puf** | 101-35-8 | ST101 | 35 | 8 | S |
| **63puf** | 007-0-8 | CJ007 | 0 | 8 | S |
| **64puf** | 007-30-8 | CJ007 | 30 | 8 | S |
| **65puf** | 007-50-8 | CJ007 | 50 | 8 | S |
| **66puf** | 009-0-8 | CJ009 | 0 | 8 | S |
| **67puf** | 009-30-8 | CJ009 | 30 | 8 | S |
| **68puf** | 009-50-8 | CJ009 | 50 | 8 | S |
| **69puf** | 009-75-8 | CJ009 | 75 | 8 | S |
| **70puf** | 009-100-8 | CJ009 | 100 | 8 | S |
| **21puf** | 101-0-11 | ST101 | 0 | 11 | A |
| **22puf** | 101-35-11 | ST101 | 35 | 11 | A |
| **23puf** | 007-0-11 | CJ007 | 0 | 11 | A |
| **24puf** | 007-30-11 | CJ007 | 30 | 11 | A |
| **25puf** | 007-50-11 | CJ007 | 50 | 11 | A |
| **26puf** | 009-0-11 | CJ009 | 0 | 11 | A |
| **27puf** | 009-30-11 | CJ009 | 30 | 11 | A |
| **28puf** | 009-50-11 | CJ009 | 50 | 11 | A |
| **29puf** | 009-75-11 | CJ009 | 75 | 11 | A |
| **30puf** | 009-100-11 | CJ009 | 100 | 11 | A |
| **51puf** | 101-0-12 | ST101 | 0 | 12 | A |
| **52puf** | 101-35-12 | ST101 | 35 | 12 | A |
| **53puf** | 007-0-12 | CJ007 | 0 | 12 | A |
| **54puf** | 007-30-12 | CJ007 | 30 | 12 | A |
| **55puf** | 007-50-12 | CJ007 | 50 | 12 | A |
| **56puf** | 009-0-12 | CJ009 | 0 | 12 | A |
| **57puf** | 009-30-12 | CJ009 | 30 | 12 | A |
| **58puf** | 009-50-12 | CJ009 | 50 | 12 | A |
| **59puf** | 009-75-12 | CJ009 | 75 | 12 | A |
| **60puf** | 009-100-12 | CJ009 | 100 | 12 | A |
| **81puf** | 101-0-1 | ST101 | 0 | 1 (2022) | W |
| **82puf** | 101-35-1 | ST101 | 35 | 1 (2022) | W |
| **83puf** | 007-0-1 | CJ007 | 0 | 1 (2022) | W |
| **84puf** | 007-30-1 | CJ007 | 30 | 1 (2022) | W |
| **85puf** | 007-50-1 | CJ007 | 50 | 1 (2022) | W |
| **86puf** | 009-0-1 | CJ009 | 0 | 1 (2022) | W |
| **87puf** | 009-30-1 | CJ009 | 30 | 1 (2022) | W |
| **88puf** | 009-50-1 | CJ009 | 50 | 1 (2022) | W |
| **89puf** | 009-75-1 | CJ009 | 75 | 1 (2022) | W |
| **90puf** | 009-100-1 | CJ009 | 100 | 1 (2022) | W |

**Table S2.** Number of reads per sample that passed through dada2 pipeline; input: number of raw reads, filtered: filtered out low quality sequences and tails, denoisedF and denoisedR: denoised sequences after the core sample inference algorithm of forward and reverse reads respectively, merged: merged sequences which are output if the forward and reverse reads overlap by at least 12 bases and are identical to each other in the overlap region, nonchim: sequences after chimera removal [1]. Samples with an unacceptably low final number of reads per sample (N<2000) were excluded from further analysis.

| **Sample_ID** | **input** | **filtered** | **denoisedF** | **denoisedR** | **merged** | **nonchim** |
| --- | --- | --- | --- | --- | --- | --- |
| 1-puf | 100636 | 91165 | 89622 | 89647 | 86631 | 81039 |
| 10-puf | 50825 | 43164 | 42631 | 42681 | 41616 | 40086 |
| 11-puf | 260963 | 189663 | 188810 | 188922 | 184065 | 173344 |
| 12-puf | 53850 | 49263 | 48824 | 48723 | 46431 | 42661 |
| 13-puf | 319178 | 260761 | 260088 | 259797 | 251482 | 240869 |
| 14-puf | 254752 | 228516 | 227760 | 227862 | 220044 | 210010 |
| 15-puf | 881 | 716 | 662 | 656 | 578 | 578 |
| 16-puf | 209071 | 155963 | 155148 | 155455 | 149734 | 130600 |
| 17-puf | 378241 | 347894 | 347191 | 346943 | 335953 | 326832 |
| 18-puf | 371509 | 345803 | 344881 | 345154 | 335980 | 325030 |
| 19-puf | 20303 | 18446 | 18141 | 18190 | 17376 | 16564 |
| 2-puf | 274369 | 244878 | 243797 | 243758 | 233445 | 219644 |
| 20-puf | 18654 | 16793 | 16538 | 16545 | 15964 | 15682 |
| 21-puf | 57081 | 51064 | 50448 | 50288 | 48156 | 45601 |
| 22-puf | 98865 | 88691 | 88029 | 87916 | 84640 | 80707 |
| 23-puf | 16447 | 14547 | 14276 | 14272 | 13652 | 13505 |
| 24-puf | 10310 | 9219 | 9055 | 9036 | 8645 | 8589 |
| 25-puf | 314520 | 270427 | 268943 | 269396 | 258414 | 244665 |
| 26-puf | 31057 | 25452 | 25130 | 25061 | 24042 | 23354 |
| 27-puf | 4477 | 3630 | 3513 | 3522 | 3353 | 3305 |
| 28-puf | 15230 | 12404 | 12186 | 12198 | 11780 | 11522 |
| 29-puf | 14546 | 11978 | 11782 | 11863 | 11468 | 11425 |
| 3-puf | 65828 | 59255 | 58330 | 58354 | 55855 | 52493 |
| 30-puf | 73144 | 61058 | 60650 | 60679 | 59206 | 58392 |
| 31-B-puf | 250477 | 218579 | 217439 | 217153 | 209142 | 185563 |
| 31-puf | 60381 | 54578 | 54148 | 54184 | 52376 | 50784 |
| 32-puf | 160666 | 145136 | 144183 | 144254 | 138348 | 127829 |
| 33-puf | 167476 | 155007 | 154301 | 154406 | 148254 | 140470 |
| 34-B-puf | 41457 | 35230 | 34821 | 34762 | 33742 | 32661 |
| 34-puf | 15961 | 14278 | 14007 | 14061 | 13710 | 13592 |
| 35-puf | 43486 | 37758 | 37441 | 37338 | 35842 | 34452 |
| 36-puf | 11867 | 10946 | 10797 | 10814 | 10606 | 10335 |
| 37-B-puf | 274421 | 232466 | 231487 | 231390 | 222762 | 198399 |
| 37-puf | 327077 | 303438 | 302478 | 302767 | 292856 | 277913 |
| 38-puf | 1390 | 1152 | 1095 | 1079 | 1027 | 1027 |
| 39-puf | 1400 | 1077 | 1007 | 964 | 938 | 934 |
| 4-puf | 93510 | 85574 | 84830 | 84975 | 81833 | 76156 |
| 40-puf | 6218 | 5318 | 5135 | 5146 | 4937 | 4927 |
| 41-puf | 83276 | 42715 | 42500 | 42283 | 40731 | 40045 |
| 42-puf | 100175 | 88254 | 87759 | 87837 | 84648 | 80705 |
| 43-puf | 96324 | 68119 | 67868 | 67914 | 65362 | 63906 |
| 44-puf | 83698 | 68018 | 67688 | 67539 | 65545 | 60377 |
| 45-puf | 131606 | 104232 | 103447 | 103109 | 98403 | 87211 |
| 46-puf | 191605 | 109811 | 109530 | 109539 | 105723 | 101952 |
| 47-puf | 255044 | 236797 | 236215 | 236172 | 229145 | 223543 |
| 48-puf | 356955 | 303569 | 302440 | 302239 | 290192 | 259244 |
| 49-puf | 57202 | 53179 | 52881 | 52787 | 51737 | 50535 |
| 5-puf | 46540 | 41341 | 40887 | 40943 | 39270 | 37729 |
| 50-puf | 37015 | 27018 | 26680 | 26536 | 25351 | 23072 |
| 51-puf | 308423 | 288306 | 287007 | 287270 | 278469 | 262756 |
| 52-puf | 234487 | 174487 | 173530 | 173379 | 166617 | 144268 |
| 53-puf | 3071 | 2255 | 2137 | 2116 | 1970 | 1878 |
| 54-puf | 145611 | 106074 | 105101 | 104761 | 100619 | 89973 |
| 55-puf | 290819 | 268368 | 267633 | 267494 | 259411 | 248629 |
| 56-puf | 20085 | 14862 | 14539 | 14559 | 13973 | 13055 |
| 57-puf | 127124 | 95576 | 95010 | 94793 | 91387 | 82971 |
| 58-puf | 7905 | 7016 | 6876 | 6881 | 6684 | 6668 |
| 59-puf | 33215 | 29000 | 28759 | 28681 | 27859 | 27441 |
| 6-puf | 162111 | 148795 | 147643 | 147779 | 142890 | 132728 |
| 60-puf | 110338 | 93756 | 93283 | 92996 | 90259 | 88680 |
| 61-puf | 8642 | 7041 | 6887 | 6874 | 6696 | 6591 |
| 62-puf | 73751 | 66402 | 65794 | 65727 | 62647 | 56953 |
| 63-puf | 11408 | 7914 | 7710 | 7649 | 7330 | 7244 |
| 64-puf | 2976 | 2451 | 2349 | 2322 | 2194 | 2180 |
| 65-puf | 116828 | 99818 | 99108 | 99177 | 95734 | 89751 |
| 66-puf | 900 | 680 | 606 | 596 | 542 | 530 |
| 67-puf | 31422 | 28306 | 28105 | 28107 | 27284 | 26228 |
| 68-puf | 48804 | 44838 | 44549 | 44564 | 43302 | 42223 |
| 69-puf | 63137 | 54755 | 54386 | 54369 | 53092 | 51940 |
| 7-puf | 205448 | 188573 | 187374 | 187393 | 181699 | 170659 |
| 70-puf | 297376 | 257988 | 256943 | 256455 | 248986 | 235200 |
| 71-puf | 27173 | 25156 | 24902 | 24879 | 23901 | 22787 |
| 72-puf | 3954 | 3410 | 3299 | 3326 | 3219 | 3176 |
| 73-puf | 1335 | 1132 | 1005 | 1015 | 948 | 941 |
| 74-puf | 22770 | 20610 | 20372 | 20339 | 19710 | 19131 |
| 75-puf | 10178 | 9114 | 8948 | 8939 | 8738 | 8671 |
| 76-puf | 8512 | 7970 | 7876 | 7831 | 7734 | 7713 |
| 77-puf | 860 | 732 | 684 | 677 | 652 | 652 |
| 78-puf | 3363 | 2966 | 2844 | 2871 | 2789 | 2776 |
| 79-puf | 140084 | 130951 | 130293 | 130160 | 125483 | 120177 |
| 8-puf | 296117 | 275820 | 274410 | 274539 | 266267 | 249038 |
| 80-puf | 66382 | 60963 | 60349 | 60481 | 58396 | 55623 |
| 81-puf | 1427 | 1194 | 1162 | 1144 | 1049 | 1032 |
| 82-puf | 3409 | 2966 | 2861 | 2865 | 2771 | 2736 |
| 83-puf | 110197 | 103178 | 102563 | 102419 | 98840 | 93685 |
| 84-puf | 83647 | 77699 | 77101 | 77090 | 74079 | 69922 |
| 85-puf | 190130 | 176050 | 175288 | 175087 | 168446 | 157088 |
| 86-puf | 2608 | 2289 | 2193 | 2167 | 2042 | 2039 |
| 87-puf | 83802 | 76530 | 76062 | 76033 | 73495 | 71415 |
| 88-puf | 236191 | 213538 | 212725 | 212591 | 205844 | 196858 |
| 89-puf | 224970 | 207151 | 206295 | 206233 | 199902 | 191598 |
| 9-puf | 242380 | 219225 | 217879 | 217801 | 210636 | 197247 |
| 90-puf | 558 | 459 | 418 | 404 | 378 | 374 |


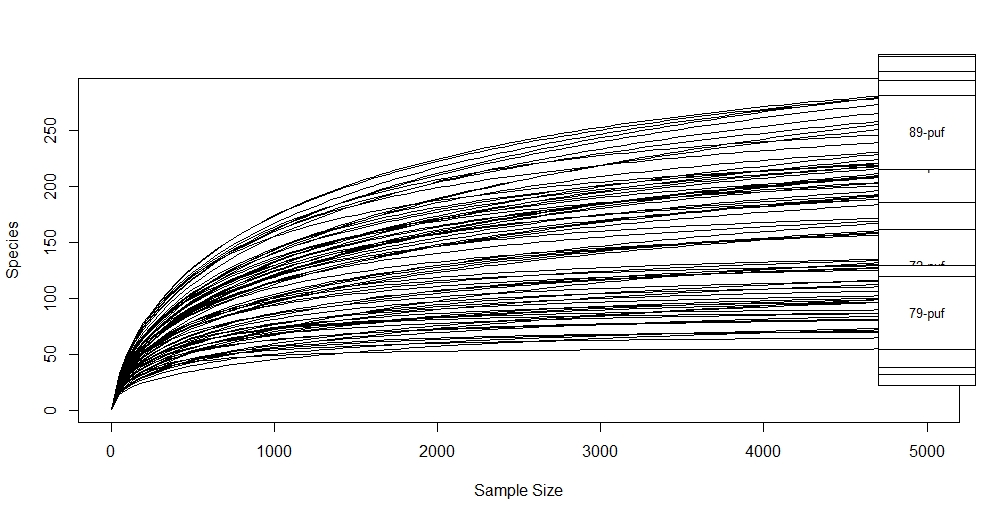


**Figure S1.** Rarefaction curve exhibiting sufficient sequencing depth (Sample Size) for AAP diversity metrics estimates. Rarefying to the smallest library size was performed multiple times (function *rarefy_even_depth* from phyloseq v1.32.0 R package [2], rarefying threshold =2000, N(times) =100).


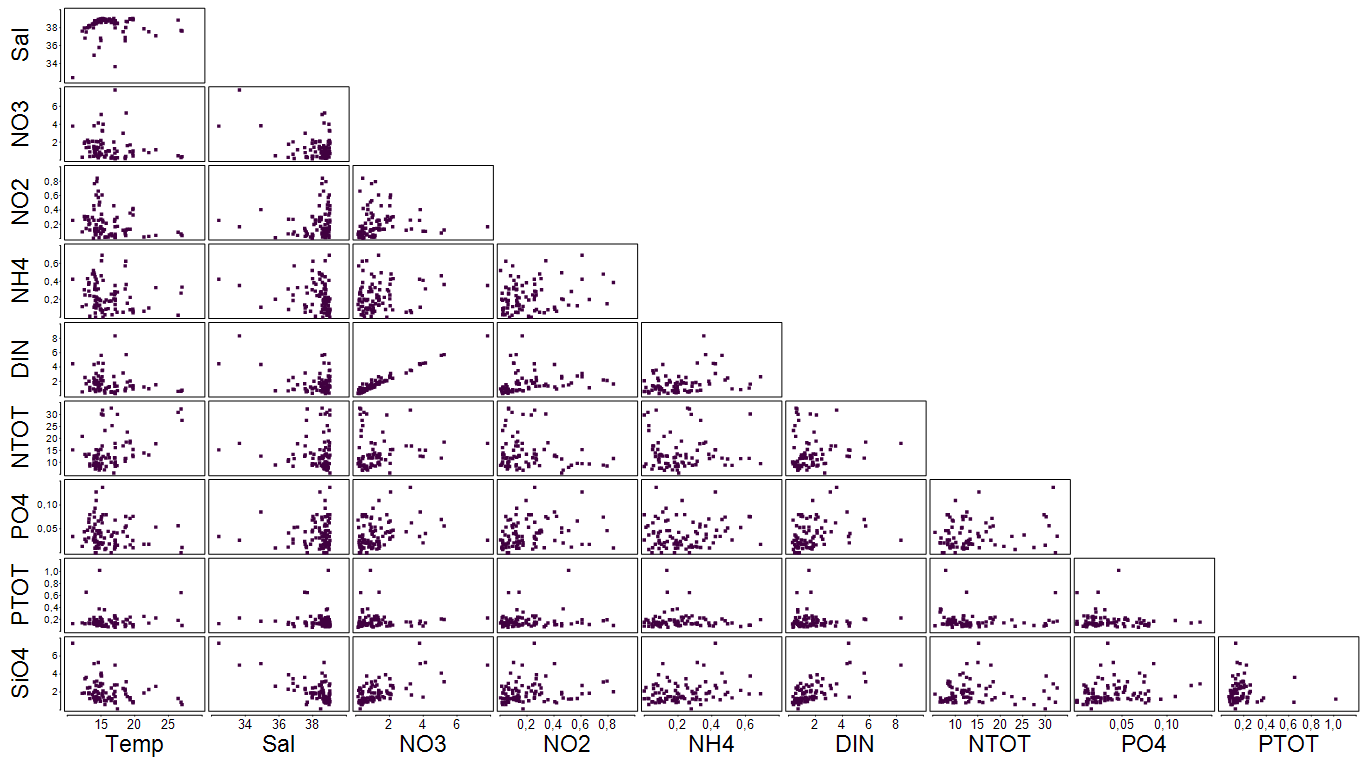


**Figure S2.** Draftsman plots used to estimate correlation of abiotic environmental variables, made in PRIMER7 [3]. Variables are: temperature-Temp, salinity-Sal, nitrates-NO3-, nitrites-NO2-, ammonium ion-NH4, dissolved inorganic nitrogen-DIN, total nitrogen-NTOT, soluble reactive phosphorus-SRP, total phosphorus-PTOT, silicate-SiO4.

**Table S3.** Permutational multivariate analysis of variance (PERMANOVA) on Euclidean distance of square-root transformed AAPs’ absolute (A) and relative (B) abundance dataset. Factors: Se- Season (fixed), Re-Region (fixed), La- Layer (nested in Region, L1 (0-30m), L2 (30-50m), L3 (50-75m), L4 (75-100m)). Pairwise comparisons for significant seasonality (W-Winter, Sp-Spring, S-Summer, A-Autumn) are given in the right part of the table. PERMANOVA was performed in PRIMER7 with 9999 permutations, unrestricted permutation of raw data, sums of squares type: Type II (conditional) [3].

A)

| **Source** | **df** | **SS** | **MS** | **Pseudo-F** | **P(perm)** | **Perms** | **Unique groups** | **t** | **P(perm)** | **Perms** |
| --- | --- | --- | --- | --- | --- | --- | --- | --- | --- | --- |
| Se | **3** | **2.593** | **0.86432** | **23.125** | **0.0001*** | **9957** | W, Sp | **5.2351** | **0.0001*** | **9840** |
| Re | 2 | 0.5968 | 0.2984 | 2.4257 | 0.0729 | 1675 | W, S | 1.7528 | 0.0825 | 9820 |
| La (Re) | 5 | 0.56493 | 0.11299 | 1.5597 | 0.1907 | 9955 | W, A | 0.55371 | 0.5794 | 9838 |
| Se x Re | 6 | 0.41104 | 0.068506 | 2.4653 | 0.0768 | 9953 | Sp, S | **7.5603** | **0.0001*** | **9833** |
| Se x La (Re) | 15 | 0.41939 | 0.02796 | 0.38595 | 0.9833 | 9911 | Sp, A | **3.9196** | **0.0006*** | **9846** |
| Res | 58 | 4.2017 | 0.072444 |  |  |  | S, A | 1.9313 | 0.0641 | 9843 |
| Total | 89 | 8.7869 |  |  |  |  |  |  |  |  |

B)

| **Source** | **df** | **SS** | **MS** | **Pseudo-F** | **P(perm)** | **Perms** | **Unique groups** | **t** | **P(perm)** | **Perms** |
| --- | --- | --- | --- | --- | --- | --- | --- | --- | --- | --- |
| Se | **3** | **7.6889** | **2.563** | **27.972** | **0.0001*** | **9948** | W, Sp | **8.914** | **0.0003*** | **9860** |
| Re | 2 | 2.368 | 1.184 | 2.131 | 0.2221 | 1675 | W, S | 1.6112 | 0.1562 | 9856 |
| La (Re) | 5 | 2.536 | 0.50719 | 2.2443 | 0.0629 | 9945 | W, A | **4.2191** | **0.0023*** | **9864** |
| Se x Re | 6 | 1.2244 | 0.20406 | 3.0069 | 0.0434 | 9952 | Sp, S | **4.8629** | **0.0044*** | **9868** |
| Se x La (Re) | 15 | 1.0297 | 0.06865 | 0.3037 | 0.9939 | 9920 | Sp, A | **6.3832** | **0.0008*** | **9840** |
| Res | 58 | 13.108 | 0.22599 |  |  |  | S, A | **3.1296** | **0.0142*** | **9868** |
| Total | 89 | 27.955 |  |  |  |  |  |  |  |  |

**Table S4.** Permutational multivariate analysis of variance (PERMANOVA) on Aitchison distance of centered-log ratio transformed *puf*M sequencing dataset agglomerated on genus-level. Factors: Se- Season (fixed), Re-Region (fixed), La- Layer (nested in Region, L1 (0-30m), L2 (30-50m), L3 (50-75m), L4 (75-100m)). Pairwise comparisons for significant seasonality (W-Winter, Sp-Spring, S-Summer, A-Autumn) are given in the right part of the table. PERMANOVA was performed in PRIMER7 with 9999 permutations, unrestricted permutation of raw data, sums of squares type: Type II (conditional) [3]

| **Source** | **df** | **SS** | **MS** | **Pseudo-F** | **P(perm)** | **Perms** | **Unique groups** | **t** | **P(perm)** | **Perms** |
| --- | --- | --- | --- | --- | --- | --- | --- | --- | --- | --- |
| Se | **3** | **1331.7** | **443.9** | **2.0572** | **0.0037*** | **9890** | W, Sp | 1.0833 | 0.2694 | 9933 |
| Re | 2 | 604.51 | 302.26 | 1.8323 | 0.0528 | 7809 | W, A | **1.4719** | **0.0156*** | **9909** |
| La(Re) | 5 | 795.15 | 159.03 | 0.70723 | 0.9791 | 9834 | W, S | **1.4807** | **0.0304*** | **9923** |
| Se x Re | 6 | 1588.8 | 264.8 | 1.3683 | 0.0703 | 9875 | Sp, A | 1.0437 | 0.3383 | 9891 |
| Se x La (Re) | 15 | 2845.1 | 189.67 | 0.84349 | 0.9247 | 9753 | Sp, S | **1.5988** | **0.0115*** | **9916** |
| Res | 49 | 11018 | 224.86 |  |  |  | A, S | **1.6526** | **0.0079*** | **9926** |
| Total | 80 | 18218 |  |  |  |  |  |  |  |  |
|  |  |  |  |  |  |  |  |  |  |  |


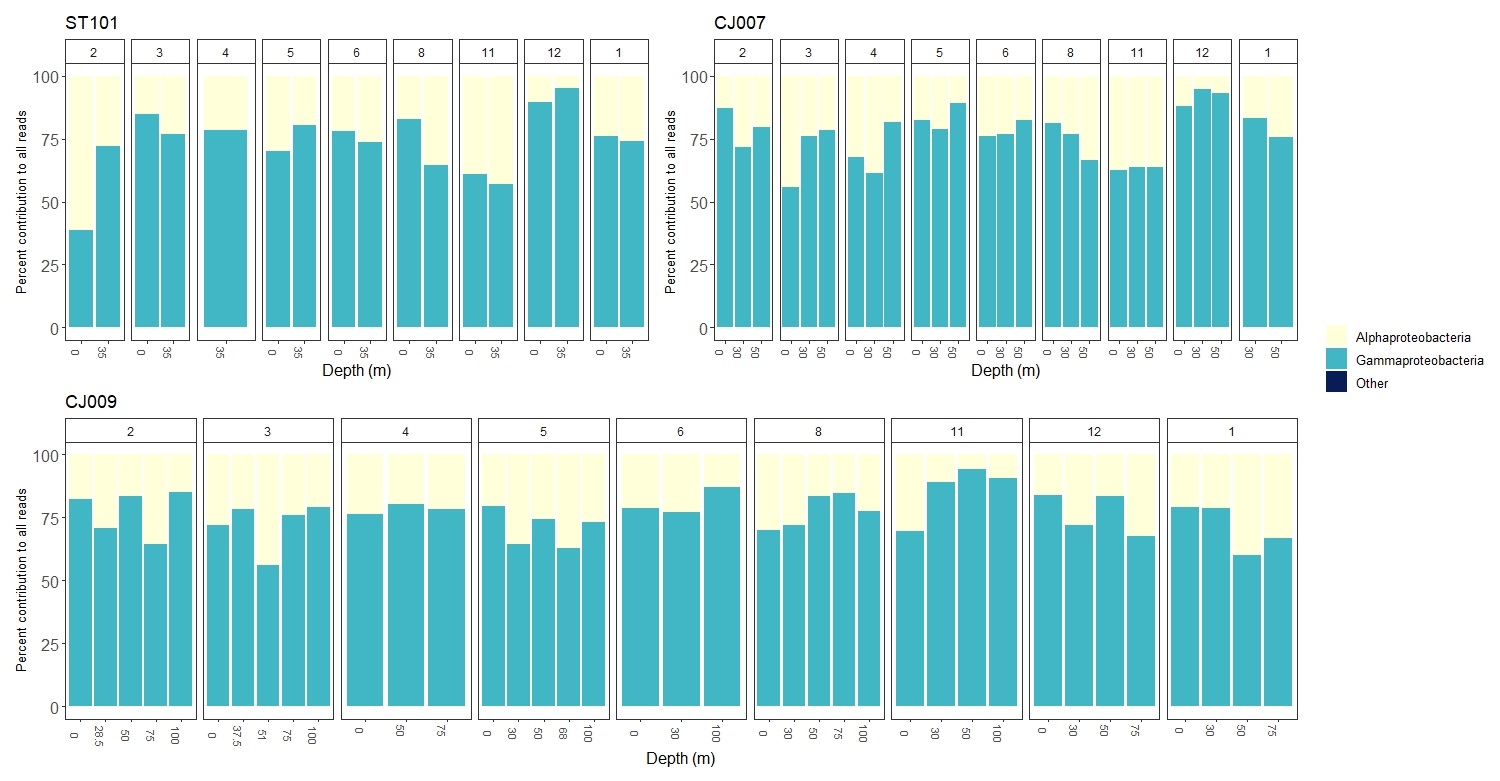


**Figure S3**. AAP community composition obtained *via* *puf*M metabarcoding in the study area shown at the class level per station (ST101, CJ007, CJ009), month and depth.

**Table S5.** Alpha diversity metrics based on rarefied *puf*M dataset (Observed number of ASVs, Shannon and Pielou’s index) given as average values with standard deviations (SD) calculated per season (vegan v2.5.7 R package [4]).

|  | **Average observed n.o of ASVs** | **SD** | **Average Shannon index** | **SD** | **Average Pielou**  **index** | **SD** |
| --- | --- | --- | --- | --- | --- | --- |
| **Winter** | 124.344 | 50.90119912 | 3.451081513 | 0.387592069 | 0.729331239 | 0.04519685 |
| **Spring** | 129.9283333 | 45.59104108 | 3.473137327 | 0.357090755 | 0.722580335 | 0.04005237 |
| **Summer** | 129.68 | 50.9395511 | 3.4993306 | 0.441423116 | 0.730711173 | 0.03868438 |
| **Autumn** | 132.6027778 | 56.37878301 | 3.393003558 | 0.682335222 | 0.701839897 | 0.07512387 |

**References**

1. Callahan BJ, McMurdie PJ, Rosen MJ, Han AW, Johnson AJA, Holmes SP. DADA2: High-resolution sample inference from Illumina amplicon data. Nat Methods. 2016;13:581–3.

2. McMurdie PJ, Holmes S. Phyloseq: An R Package for Reproducible Interactive Analysis and Graphics of Microbiome Census Data. PLoS One. 2013;8.

3. Clarke KR, Gorley RN. Primer: User manual/tutorial. Prim Ltd, Plymouth, UK. 2015;:93.

4. Oksanen AJ, Blanchet FG, Friendly M, Kindt R, Legendre P, Mcglinn D, et al. vegan community ecology package version 2.5-7 2020
