# Supplementary material for: Ecology of aerobic anoxygenic phototrophs on a fine-scale taxonomic resolution in Adriatic Sea unravelled by unsupervised neural network
Source: Environ Microbiome. 2024 Apr 29;19:28. doi: 10.1186/s40793-024-00573-6 (PMC11059731; doi:10.1186/s40793-024-00573-6)
Supplement: Supplementary file 2 — Additional file 2. Detailed description of Infrared epifluorescence microscopy and fluorescence in situ hybridisation (FISH-IR). Table S1. Permutational multivariate analysis of variance (PERMANOVA) on Bray-Curtis similarity for square-root transformed FISH-IR relative abundance dataset. Factors: Se- Season (fixed), Re-Region (fixed), La- Layer (nested in Region, L1 (0-30m), L2 (30-50m), L3 (50-75m), L4 (75-100m)). Pairwise comparisons for significant seasonality (W-Winter, Sp-Spring, S-Summer, A-Autumn) are given in the right part of the table. PERMANOVA was performed in PRIMER7 with 9999 permutations, Unrestricted permutation of raw data, sums of squares type: Type II (conditional) [5]., Figure S1. Two-dimensional nonmetric multidimensional scaling (NMDS) ordination plot of FISH-IR square root transformed dataset (relative abundances of AAPs assigned to Alphaproteobacteria, Gammaproteobacteria and Roseobacter clade) on a seasonal scale (W-Winter, Sp-Spring, S-Summer, A-Autumn)., Figure S2. Spatio-temporal distribution of FISH-IR groups (probes for Alphaproteobacteria, Gammaproteobacteria, Roseobacter) given as relative abundances of AAPs (n=90 samples), shown per station (ST101, CJ007 and CJ009), month, and depth., Figure S3. Seasonal distribution (W-winter, Sp-Spring, S-Summer, A-Autumn) of FISH-IR groups (Alphaproteobacteria, Gammaproteobacteria, Roseobacter) given as average relative abundances of AAPs. [file 40793_2024_573_MOESM2_ESM.docx]

**Additional file 2**

**Ecology of Aerobic Anoxygenic Phototrophs on a fine-scale taxonomic resolution in Adriatic Sea unravelled by unsupervised neural network**

Iva Stojan^1,2^, Danijela Šantić^1^*, Cristian Villena-Alemany^3,4^, Željka Trumbić^5^, Frano Matić^5^, Ana Vrdoljak Tomaš^1^, Ivana Lepen Pleić^1^, Kasia Piwosz^6^, Grozdan Kušpilić^1^, Živana Ninčević Gladan ^1^, Stefanija Šestanović^1^, Mladen Šolić^1^

^1^ Institute of Oceanography and Fisheries, Šetalište Ivana Meštrovića 63, Split, Croatia

^2^ Doctoral Study of Biophysics, Faculty of Science, University of Split, Ruđera Boškovića 37, Split, Croatia

^3^ Laboratory of Anoxygenic Phototrophs, Institute of Microbiology, Czech Academy of Sciences, 37981 Třeboň, Czechia

^4^ Department of Ecosystem Biology, Faculty of Science, University of South Bohemia, České Budějovice, Czechia

^5^ University Department of Marine Studies, University of Split, Ruđera Boškovića 37, Split, Croatia

^6^ Department of Fisheries, Oceanography and Marine Ecology, National Marine Fisheries Research Institute, Gdynia, Poland

*Danijela Šantić, Institute of Oceanography and Fisheries, Šetalište Ivana Meštrovića 63, Split, Croatia, e-mail: [segvic@izor.hr](mailto:segvic@izor.hr)

**Number of pages: 7**

**Number of tables: 1**

**Number of figures: 3**

## **Infrared epifluorescence microscopy and fluorescence *in situ* hybridisation (FISH-IR)**

**Methods**

After fixation of marine samples with formaldehyde (pH 7.5, f.c. 2%) for 1 h at room temperature or overnight at 4 ⁰C, samples were filtered onto white 0.2 µm polycarbonate filters (47 mm diameter, Whatman® Nuclepore™ Track-Etched, Merck), which were washed with Mili-Q water and stored immediately after filtration at −20°C until further analysis. Fluorescent oligonucleotide probes double-labelled with FITC at 3’ and 5’ ends (Biomers, Germany) targeting Alphaproteobacteria (probe ALF968), Gammaproteobacteria (probes GAM42a and unlabelled Bet42a competitor) and the *Roseobacter* clade (probe ROS537) were hybridised with the samples at 35°C for 150 min (f. c. 45% formamide in the hybridisation buffer for probe ALF968 and 55% formamide for probes GAM42a and ROS37) [1–3]. After hybridisation, samples were washed at 37 ⁰C for 20 min and rinsed with Mili-Q water and never ethanol [3]. After drying, samples were counterstained with DAPI (f. c. 1 µgmL^-1^) using a 3:1 mixture of Citifluor™ AF1 and Vectashield®. Enumeration was performed using an Olympus BX51 epifluorescence microscope equipped with an Olympus UPlanSApo 100×/1.40 OIL, IR objective, a U-LH100H6 Hg lamp and CellSens software for image analysis. Because of rapid Bchl *a* autofluorescence fading, four epifluorescent filter sets were applied in a specific order: IR, FITC, DAPI and Chl *a*. The Chl *a* signal was subtracted from IR to obtain a net count of AAP cells, while at the same time the signals from the hybridised fluorescent probes were recorded for each AAP cell. For each sample, 10 to 12 images (400 to 600 DAPI cells) randomly selected across the filter sections were acquired and analysed. The relative abundance of each group was calculated as the percentage (%) of total AAP counts.

Aditionally, in silico specificity (*i.e.* number of matching rRNA sequences outside the target taxon) and coverage (*i.e*. percentage of matching rRNA sequences within the target taxon) of ALF968 and ROS537 probes were estimated against SILVA138 SSU [4] database (accessed in October 2023) with TestProbe 3.0 to obtain taxa targeted by these probes, since ALF968 covers numerous alphaproteobacterial orders, including Rhodobacterales (target of ROS537 probe) (Additional file 9).

**Results**

Counts are presented as relative abundances (*i.e.* percentage of AAPs). As with AAP absolute abundances, seasonality was significant according to nMDS and PERMANOVA based on Bray-Curtis distances of square-root transformed dataset (pseudo-F= 4.3159, p=0.004, unique permutations=9960). However, there were insignificant differences observed in terms of depth and region factors (Table S1 and Figure S1). Pairwise comparisons showed that summer was the only season that differed from the others. Insignificant result of PERMDISP (group factor: Season, F=0.44762, p=0.749, 9999 permutations) indicated that groups did not differ in dispersion.

**Table S1**. Permutational multivariate analysis of variance (PERMANOVA) on Bray-Curtis similarity for square-root transformed FISH-IR relative abundance dataset. Factors: Se- Season (fixed), Re-Region (fixed), La- Layer (nested in Region, L1 (0-30m), L2 (30-50m), L3 (50-75m), L4 (75-100m)). Pairwise comparisons for significant seasonality (W-Winter, Sp-Spring, S-Summer, A-Autumn) are given in the right part of the table. PERMANOVA was performed in PRIMER7 with 9999 permutations, Unrestricted permutation of raw data, sums of squares type: Type II (conditional) [5].

| **Source** | **df** | **SS** | **MS** | **Pseudo-F** | **P(perm)** | **Perms** | **Unique groups** | **t** | **P(perm)** | **Perms** |
| --- | --- | --- | --- | --- | --- | --- | --- | --- | --- | --- |
| Se | **3** | **1226.2** | **408.74** | **4.3159** | **0.004*** | **9960** | W, Sp | 1.7217 | 0.0808 | 9964 |
| Re | 2 | 213.71 | 106.86 | 0.88152 | 0.5378 | 4310 | W, S | **3.2674** | **0.0023*** | **9967** |
| La(Re) | 5 | 579.64 | 115.93 | 1.7652 | 0.0703 | 9934 | W, A | 1.499 | 0.1603 | 9958 |
| Se x Re | 6 | 482.88 | 80.481 | 1.0677 | 0.4218 | 9937 | Sp, S | **2.4999** | **0.0103*** | **9958** |
| Se x La (Re) | 14 | 1041.4 | 74.387 | 1.1326 | 0.3086 | 9900 | Sp, A | 1.9106 | 0.062 | 9962 |
| Res | 58 | 3809.2 | 65.676 |  |  |  | S, A | **2.5567** | **0.0145*** | **9956** |
| Total | 88 | 7367.2 |  |  |  |  |  |  |  |  |


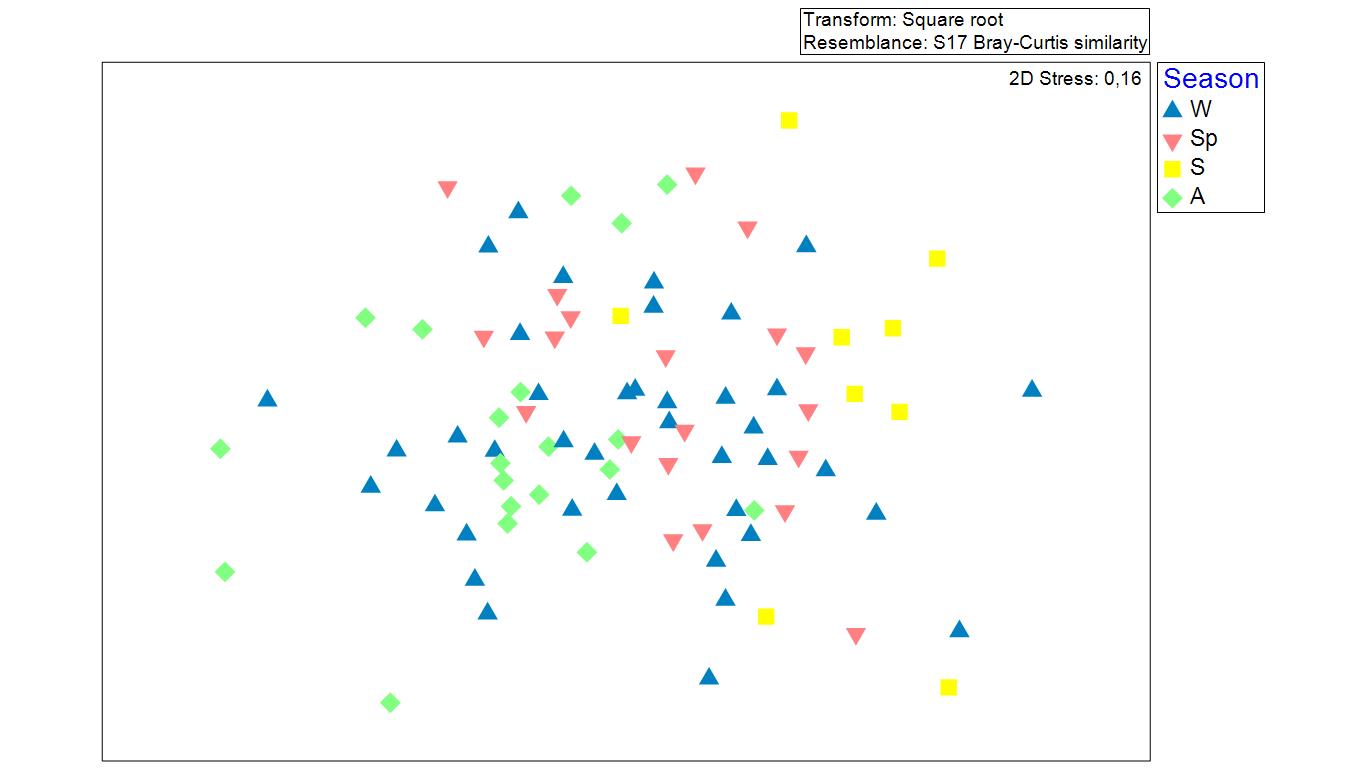


**Figure S1**. Two-dimensional nonmetric multidimensional scaling (NMDS) ordination plot of FISH-IR square root transformed dataset (relative abundances of AAPs assigned to Alphaproteobacteria, Gammaproteobacteria and *Roseobacter* clade) on a seasonal scale (W-Winter, Sp-Spring, S-Summer, A-Autumn).

In the study area, gammaprotebacterial AAPs had a mean relative abundance of 35.25%, Alphaproteobacteria 31.15% and *Roseobacter* 37.66%.

At the individual sample level, Alphaproteobacteria AAPs had the highest relative abundance of ~85% in February at 75 m depth at open ocean station CJ009, followed by the second highest relative abundance (~70%) in March at 100 m depth at the same station. In contrast, the lowest abundances (7%) were recorded at station ST101 at 35 m depth in December and at station CJ009 at 100 m depth in November (Figure S2). Regarding seasonal distribution, Alphaproteobacteria AAPs showed generally higher abundances in summer, and lowest ones in autumn (Figure S3).

AAPs belonging to *Roseobacter* clade showed the highest relative abundance (77.77%) at station ST101 at 35 m in August and the lowest one (14.29%) at CJ007 station at 50 m in August (Figure S2). In terms of a season, an inverse pattern was observed to that of the general Alphaproteobacteria. The highest average relative abundances were recorded in autumn and the lowest average ones in summer, except for ST101 station (Figure S3).

AAPs belonging to the class of Gammaproteobacteria had the highest relative abundances in samples collected from the surface (0 m) of open sea station CJ009 in February (78.9%), followed by samples from station CJ007 at 30 m depth in August (75%) and station ST101 at 35 m depth also in August (71.4%) (Figure S2). The lowest value (8.3%) was measured at the sea surface in January at station CJ009. In terms of seasonal distribution, they were on average predominant in summer at all stations, with the lowest contribution in winter (Figure S3).


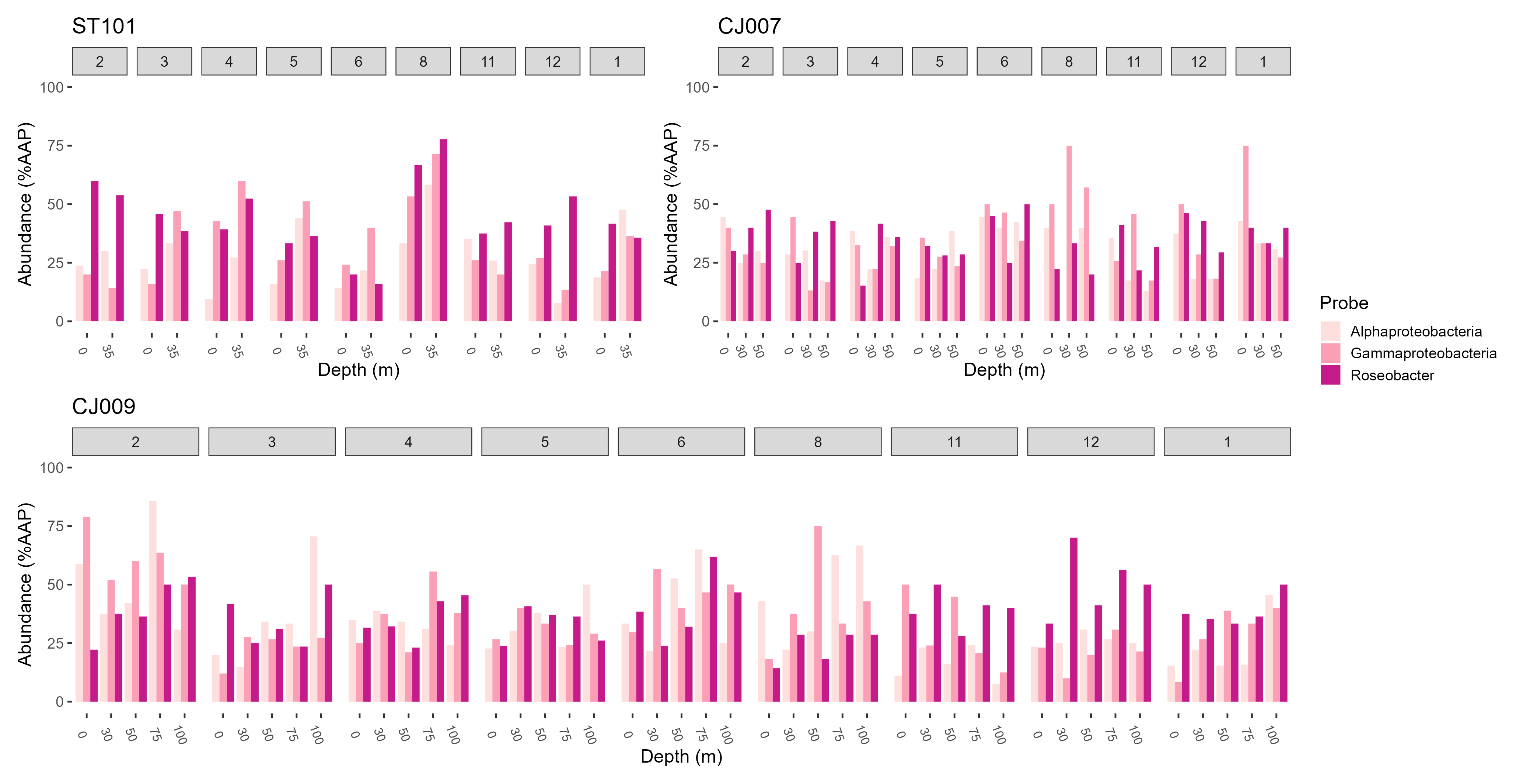


**Figure S2**. Spatio-temporal distribution of FISH-IR groups (probes for Alphaproteobacteria, Gammaproteobacteria, *Roseobacter*) given as relative abundances of AAPs (n=90 samples), shown per station (ST101, CJ007 and CJ009), month, and depth.


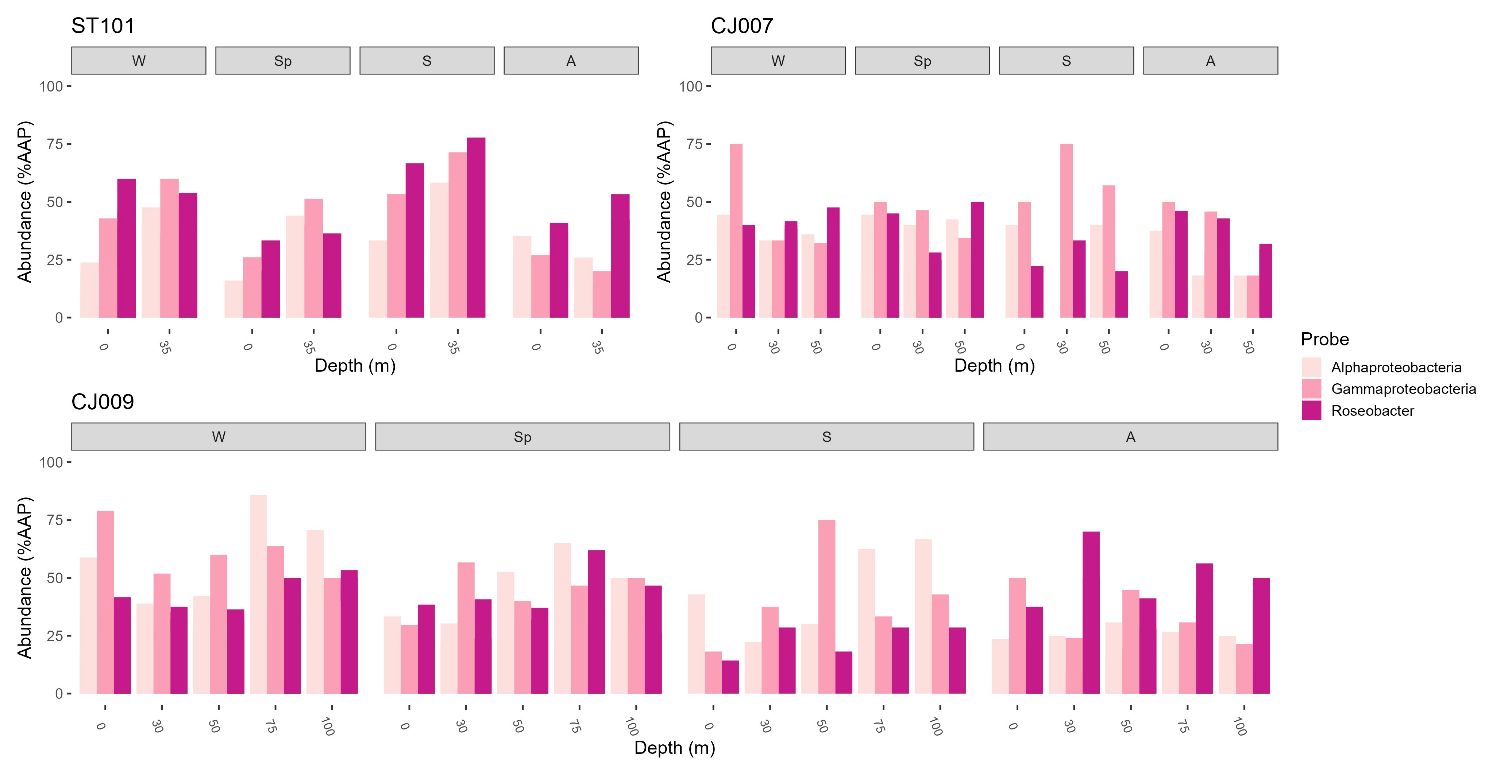


**Figure S3.** Seasonal distribution (W-winter, Sp-Spring, S-Summer, A-Autumn) of FISH-IR groups (Alphaproteobacteria, Gammaproteobacteria, *Roseobacter*) given as average relative abundances of AAPs.

**References**

1. Manz W, Amann R, Ludwig W, Wagner M, Schleifer KH. Phylogenetic Oligodeoxynucleotide Probes for the Major Subclasses of Proteobacteria: Problems and Solutions. Syst Appl Microbiol. 1992;15:593–600.

2. Eilers H, Pernthaler J, Peplies J, Glöckner FO, Gerdts G, Amann R. Isolation of Novel Pelagic Bacteria from the German Bight and Their Seasonal Contributions to Surface Picoplankton. Appl Environ Microbiol. 2001;67:5134–42.

3. Kasalický V, Zeng Y, Piwosz K, Šimek K, Kratochvilová H, Koblížek M. Aerobic anoxygenic photosynthesis is commonly present within the genus Limnohabitans. Appl Environ Microbiol. 2018;84.

4. Quast C, Pruesse E, Yilmaz P, Gerken J, Schweer T, Yarza P, et al. The SILVA ribosomal RNA gene database project: Improved data processing and web-based tools. Nucleic Acids Res. 2013;41:590–6.

5. Clarke KR, Gorley RN. Primer: User manual/tutorial. Prim Ltd, Plymouth, UK. 2015;:93.
