# Supplementary material for: Ecology of aerobic anoxygenic phototrophs on a fine-scale taxonomic resolution in Adriatic Sea unravelled by unsupervised neural network
Source: Environ Microbiome. 2024 Apr 29;19:28. doi: 10.1186/s40793-024-00573-6 (PMC11059731; doi:10.1186/s40793-024-00573-6)
Supplement: Supplementary file 6 — Additional file 6. Spatio-temporal distribution of Luminiphilus ASVs. Figure 1. Genus Luminiphilus composition via pufM metabarcoding at ST101 station shown per month and depth. Category “Other” represents ASVs with relative abundances below 5%., Figure 2. Genus Luminiphilus composition via pufM metabarcoding at CJ007 station shown per month and depth. Category “Other” represents ASVs with relative abundances below 5%., Figure 3. Genus Luminiphilus composition via pufM metabarcoding at CJ009 station shown per month and depth. Category “Other” represents ASVs with relative abundances below 5%. [file 40793_2024_573_MOESM6_ESM.docx]

**Additional file 6**

**Ecology of Aerobic Anoxygenic Phototrophs on a fine-scale taxonomic resolution in Adriatic Sea unravelled by unsupervised neural network**

Iva Stojan^1,2^, Danijela Šantić^1^*, Cristian Villena-Alemany^3,4^, Željka Trumbić^5^, Frano Matić^5^, Ana Vrdoljak Tomaš^1^, Ivana Lepen Pleić^1^, Kasia Piwosz^6^, Grozdan Kušpilić^1^, Živana Ninčević Gladan ^1^, Stefanija Šestanović^1^, Mladen Šolić^1^

^1^ Institute of Oceanography and Fisheries, Šetalište Ivana Meštrovića 63, Split, Croatia

^2^ Doctoral Study of Biophysics, Faculty of Science, University of Split, Ruđera Boškovića 37, Split, Croatia

^3^ Laboratory of Anoxygenic Phototrophs, Institute of Microbiology, Czech Academy of Sciences, 37981 Třeboň, Czechia

^4^ Department of Ecosystem Biology, Faculty of Science, University of South Bohemia, České Budějovice, Czechia

^5^ University Department of Marine Studies, University of Split, Ruđera Boškovića 37, Split, Croatia

^6^ Department of Fisheries, Oceanography and Marine Ecology, National Marine Fisheries Research Institute, Gdynia, Poland

*Danijela Šantić, Institute of Oceanography and Fisheries, Šetalište Ivana Meštrovića 63, Split, Croatia, e-mail: [segvic@izor.hr](mailto:segvic@izor.hr)

**Number of pages: 4**

**Number of figures: 3**

In total, 146 *Luminiphilus* ASVs were detected in the study area with specific variants present in different relative abundances at each station. Hence, *Luminiphilus* composition is shown separately in Figures S1 for station ST101, S2 for station CJ007 and S3 for station CJ009 with brief descriptions of ASV trends given below.


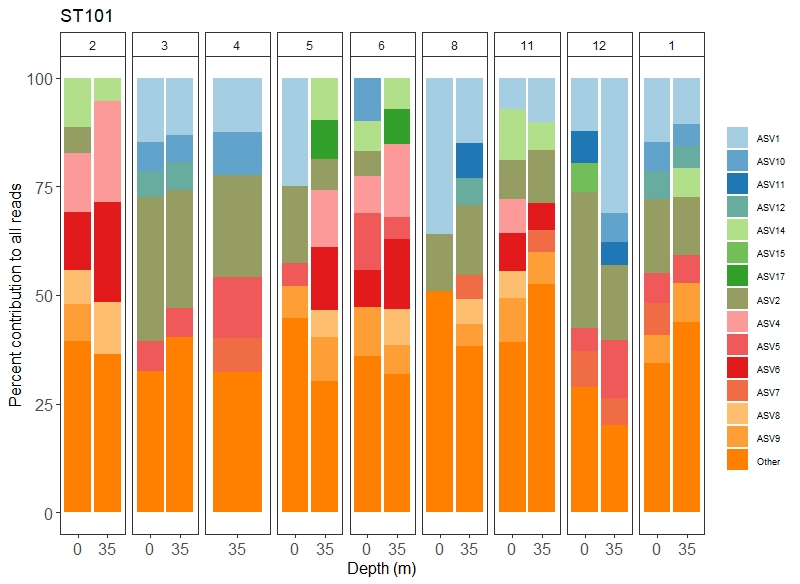


**Figure 1.** Genus *Luminiphilus* composition *via puf*M metabarcoding at ST101 station shown per month and depth. Category “Other” represents ASVs with relative abundances below 5%.

At coastal station ST101, ASV1 was present in relative abundances higher than 5% in all samples except those from February and June (0 and 35 m) and one sample collected in May at sea-bottom. It had the highest contribution of ~30% in sample from August (0 m) and December (35 m). ASV4 had the highest relative abundances in February and June at sea-surface and bottom as well as in May at sea bottom. ASV6 had higer values at sea-surface and bottom in months of February, June and November as well as in May (35 m). AVS14 was present in higher relative abundances in February, June and November at both depths as well as May and January at sea-bottom. On the other hand, ASV2 was present in all samples in abundances higher than 5%, except in February and June (35 m). ASV10 was detected in higher relative abundances in March (both depths), April (35 m), June (0 m), December (35 m) and January (both depths) while ASV11 was detected in relative abundances greater than 5% in August (35 m) and December (0 and 35 m). Numerus other ASVs were present in relative abundances below 5%.


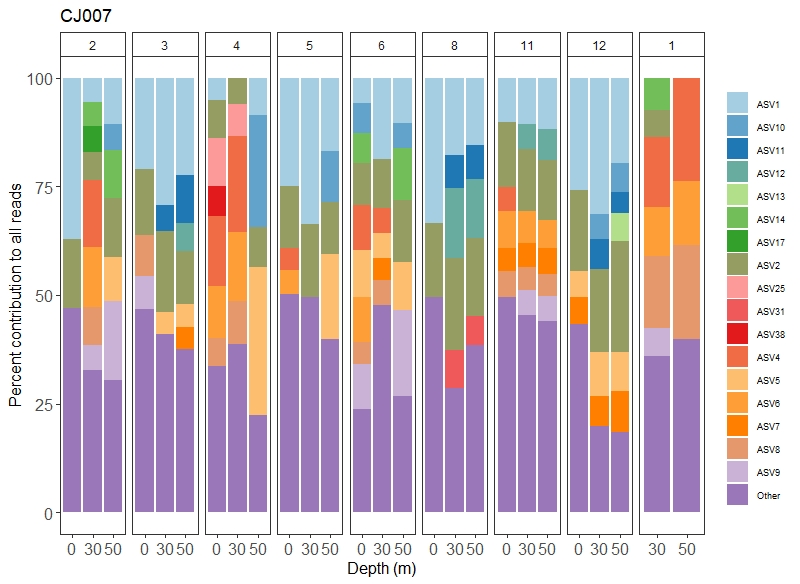
**Figure 2.** Genus *Luminiphilus* composition *via puf*M metabarcoding at CJ007 station shown per month and depth. Category “Other” represents ASVs with relative abundances below 5%.

At transitional station CJ007, ASV1 was present at all depths and months with relative contribution up to ~40% except in April (30 m) and January (30 and 50 m) when it's contribution dropped below 5%. ASV11 was detected in higher relative contribution at depths 30 and 50 m in March, August and December. ASV10 had relative abundance of ~20% in April at depth of 50 m. ASV2 was omnipresent in all samples in relative abundances greater than 5% except in sample from January at depth of 50 m. ASV38 has higher contribution in April at sea-surface, while ASV25 appeared in abundances ~10% also in April at 0 and 35 m. ASV9 had relative abundances greater than 5% in February (30 and 50 m), March (0 m), June (0 and 50 m), November (30 and 50 m) and January (0 m). ASV4 had the highest relative abundances in February (30 m), April (0 and 30 m) and January (30 and 50 m), while relative contribution of ASV12 and ASV31 was greatest in August at depths of 30 and 50 m. ASV8 had the highest relative abundances in January (depths 30 and 50 m).


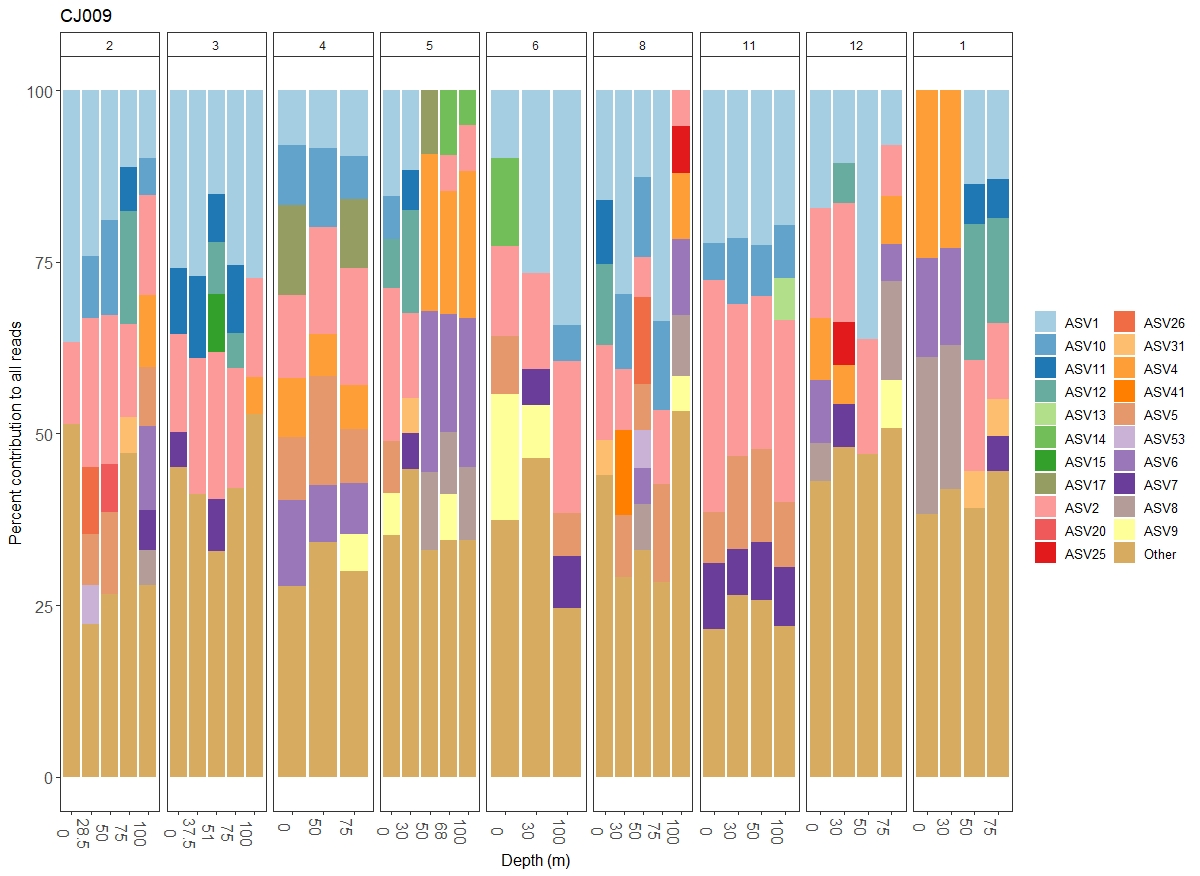


**Figure 3.** Genus *Luminiphilus* composition *via puf*M metabarcoding at CJ009 station shown per month and depth. Category “Other” represents ASVs with relative abundances below 5%.

Open sea station CJ009, in comparison to other stations, had the highest contribution of rare ASVs (relative abundances below 5%), exceeding 50% in some samples. ASV2 had the highest relative abundances at all depths in February, March, April, June, November and December, while relative abundances below 5% were recorded in January at 0 and 30 m. ASV7 had the highest contribution at all depths in November with occasional peaks at different depths in February (100 m), March (0 and 51 m), May (30 m), June (30 and 100 m), December (30 m) and January (75 m). ASV9 had the highest relative contribution at sea-surface in June. ASV25 was recorded in higher relative abundance in August (100 m) and December (30 m), while ASV20 had relative abundance above 5 % in February (28.5 and 50 m). ASV8 had the highest relative contribution in samples from January (0 and 30 m), while ASV6 dominated together with ASV4 in May at depths of 50, 68 and 100 m and January at depths 0 and 30 m. AVS 17 had greater relative contribution in April at depths of 0 and 75 m, while ASV5 had greater relative abundances in April and November at all depths.
