# Supplementary material for: Ecology of aerobic anoxygenic phototrophs on a fine-scale taxonomic resolution in Adriatic Sea unravelled by unsupervised neural network
Source: Environ Microbiome. 2024 Apr 29;19:28. doi: 10.1186/s40793-024-00573-6 (PMC11059731; doi:10.1186/s40793-024-00573-6)
Supplement: Supplementary file 7 — Additional file 7. Detailed description of neural gas analysis results. Figure S1. Heatmap of order-agglomerated and CLR-transformed pufM dataset resulting in five distinct BMUs. Samples are shown for each unit., Figure S2. Genus-agglomerated and CLR-transformed pufM dataset resulting in five distinct BMUs. Samples are shown for each BMU., Figure S3. Neural gas analysis results of pufM dataset at clr-transformed ASV-level, clustered into five BMUs. Biotic (UHB, HIGH, SYN, PROCHL, PE, BP, HNF, AAP) and abiotic (Temp, Sal, NO3-, NO2-, NH4+, DIN, NTOT, PO43-, PTOT, SiO42-, Chl a) variables of each environment are given in (A) as average value for each unit. Colour gradient from red to green represents the lowest and highest average values respectively. Relative contribution expressed as CLR-transformed value of specific ASV is shown in (B). ASVs are ordered by genus, with most dominant genera ordered alphabetically. [file 40793_2024_573_MOESM7_ESM.docx]

**Additional file 7**

**Ecology of Aerobic Anoxygenic Phototrophs on a fine-scale taxonomic resolution in Adriatic Sea unravelled by unsupervised neural network**

Iva Stojan^1,2^, Danijela Šantić^1^*, Cristian Villena-Alemany^3,4^, Željka Trumbić^5^, Frano Matić^5^, Ana Vrdoljak Tomaš^1^, Ivana Lepen Pleić^1^, Kasia Piwosz^6^, Grozdan Kušpilić^1^, Živana Ninčević Gladan ^1^, Stefanija Šestanović^1^, Mladen Šolić^1^

^1^ Institute of Oceanography and Fisheries, Šetalište Ivana Meštrovića 63, Split, Croatia

^2^ Doctoral Study of Biophysics, Faculty of Science, University of Split, Ruđera Boškovića 37, Split, Croatia

^3^ Laboratory of Anoxygenic Phototrophs, Institute of Microbiology, Czech Academy of Sciences, 37981 Třeboň, Czechia

^4^ Department of Ecosystem Biology, Faculty of Science, University of South Bohemia, České Budějovice, Czechia

^5^ University Department of Marine Studies, University of Split, Ruđera Boškovića 37, Split, Croatia

^6^ Department of Fisheries, Oceanography and Marine Ecology, National Marine Fisheries Research Institute, Gdynia, Poland

*Danijela Šantić, Institute of Oceanography and Fisheries, Šetalište Ivana Meštrovića 63, Split, Croatia, e-mail: [segvic@izor.hr](mailto:segvic@izor.hr)

**Number of pages: 8**

**Number of figures: 3**

**DETAILED DESCRIPTION OF NEURAL GAS ANALYSIS RESULTS**

**ORDER-LEVEL NEURAL GAS ANALYSIS**

BMU1 (34.57% of the data) appeared to be warm and nutrient-enriched environment with the lowest average salinity (37.96) and temperature of 16.43 °C. This was the largest unit gathering 28 samples. The highest values of AAP Gammaproteobacteria of the order Burkholderiales and the alphaproteobacterial lineage SP197 were observed. On the other hand, orders Rhizobiales, Sphingomonadales, Caulobacterales and Arenicellales occurred in this unit with lower contribution. Environmental factors representing this unit were the highest average absolute abundances of AAPs, total heterotrophic bacteria, Synechococcus and HNF combined with the highest recorded concentrations of nitrates, dissolved inorganic nitrogen, phosphates, N/P ratio and high Chl a. As for FISH-IR counts, the Roseobacter clade had the highest relative abundances in this unit, while AAPs classified as Gammaproteobacteria had the lowest. Neither clear seasonal nor regional patterns in samples were observed in this unit.

BMU2 (19.75% of the data) was characterised by the highest average temperature measured (16.63 °C) and an increased occurrence of the orders Rhizobiales, Sphingomonadales, Caulobacterales and Arenicellales. This unit was the shallowest one with an average depth of 23.4 m and did not represent a clear spatial-seasonal pattern. HNA bacteria had the highest absolute abundances in this unit, along with high abundances of Synechococcus. Nitrates had the lowest average concentrations, while Chl a had the highest. All FISH-IR groups had similar contributions.

BMU3 (14.81% of the data) was similar to BMU2 in that the AAP orders Rhizobiales, Sphingomonadales, Caulobacterales and Arenicellales had the highest values in this unit. The average temperature in this unit was above 16 °C. The decisive biological factor describing this unit was the lowest bacterial production. The ammonia concentration was lowest in this unit. As for FISH-IR, both AAPs belonging to the Gammaproteobacteria and Alphaproteobacteria classes had the highest average abundances, while in contrast, the Roseobacter clade had the lowest ones.

BMU4 (22.22% of the data), in contrast to BMU2 and BMU3, had the lowest abundances of the orders Rhizobiales, Sphingomonadales, Caulobacterales and Arenicellales, which again showed the same pattern of occurrence. This unit was characterised by an average temperature of 15.5 °C and maximum nitrite concentrations. The average depth was ~42 m with the highest absolute abundance of Prochlorococcus and picoeukaryotes. FISH-IR counts revealed the lowest relative abundance of alphaproteobacterial AAPs in this unit.

BMU5 (8.64% of the data) was described with the lowest number of 7 samples, indicating that this was the rarest environment described with this model. This unit represented the coldest, scarcest and deepest environment, with an average temperature of 15°C and an average depth of 57.9 m. Salinity reached record values of 38.71. Interestingly, the AAP order UBA8366 had the highest values only in this unit, as did an unclassified order belonging to the Alphaproteobacteria class. The lowest abundances of heterotrophic bacteria, Synechococcus, Prochlorococcus, picoeukaryotes, HNF and AAPs were found in this unit. This is the scarcest environment with the lowest concentrations of nitrites and nitrates, dissolved inorganic nitrogen, phosphorus, silicates, Chl a and the lowest N/P ratio. However, ammonia concentration and bacterial production reached their maxima in this unit. As for the results of FISH-IR, both Roseobacter and Gammaproteobacterial AAPs showed high relative abundances. This unit gathered stations towards the open sea (CJ007 and CJ009), with no clear seasonality observed.


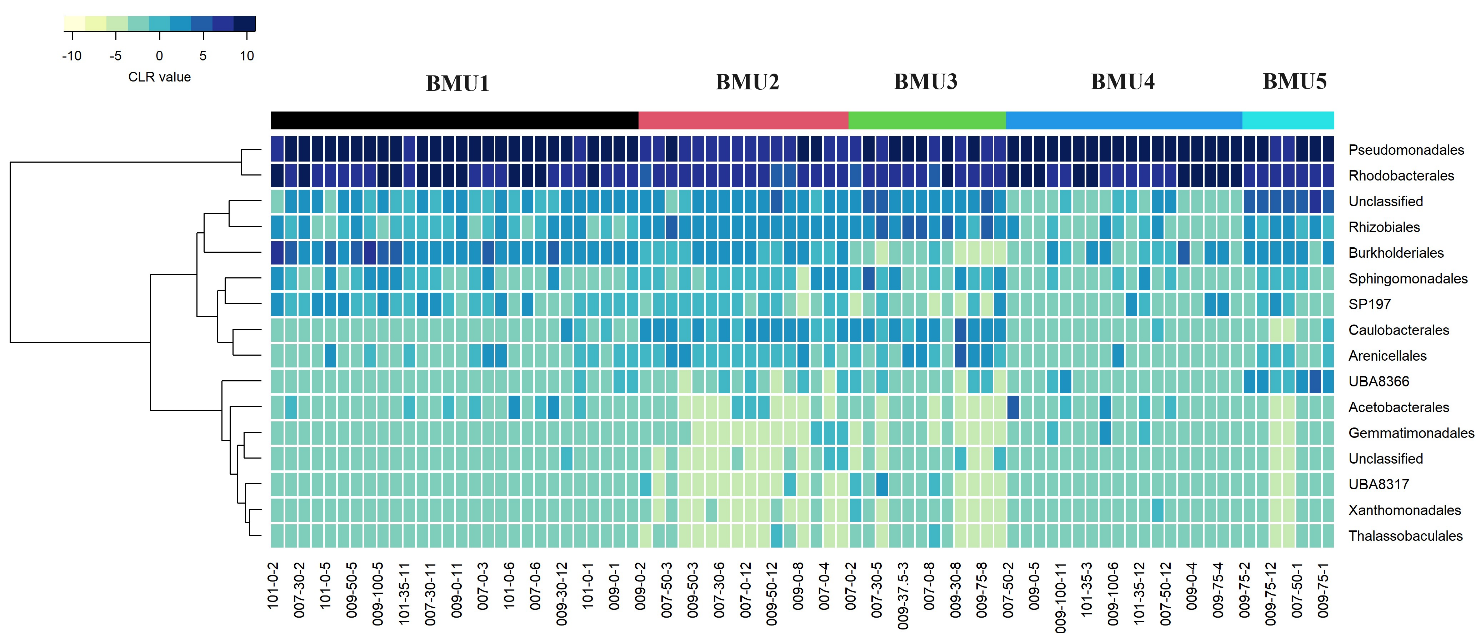


**Figure S1.** Heatmap of order-agglomerated and CLR-transformed *puf*M dataset resulting in five distinct BMUs. Samples are shown for each unit.

**GENUS-LEVEL NEURAL GAS ANALYSIS**

BMU1 (13.58% of the data) was a nutrient-enriched environment with a minimum average salinity (37.568). In contrast, nitrates, ammonia, dissolved inorganic nitrogen, phosphorus and silicates had the highest average concentrations in the samples describing this unit. Absolute abundances of AAPs had the highest values in this unit, as did absolute counts of total heterotrophic bacteria and picoeukaryotes. On the opposite, bacterial production and abundances of HNF were lowest in this unit. Diversity indices, the observed number of ASVs and the Shannon index reached their highest values in this unit. Some of AAP genera that reached their maxima in this unit were *Planktomarina*, *Nereida*, *CYK10* and *Thalassobacter*, all belonging to the Rhodobacteraceae family (Alphaproteobacteria); and *RS62*, *Rhodoferax* and *Limnohabitans* from the Burkholderiaceae family (Gammaproteobacteria). No seasonal nor regional patterns were observed in this unit.

BMU2 (22.22% of the data) was characterised by the highest recorded average temperature of all units (16.93 °C). No distinct spatiotemporal patterns were observed in this unit. Ammonia and Chl *a* had minimum values in this unit, as well as low concentrations of nitrates and nitrites. In terms of biotic variables, the lowest abundances of heterotrophic bacteria, *Synechococcus*, picoeukaryotes and low abundances of AAPs and nanoflagellates were observed in this environment. AAP genera that reached their average maximum values were *Erythrobacter* from the family Sphingomonadaceae (Sphingomonadales, Alphaproteobacteria), an unclassified genus from the family Xanthobacteraceae (Rhizobiales, Alphaproteobacteria), two unclassified genera of the unknown family belonging to the order Rhizobiales, and *Planktotalea* and *Palleronia* from the family Rhodobacteraceae (Alphaproteobacteria).

BMU3 (20.99% of the data) was defined with the highest average salinity (38.48). Other abiotic factors pronounced in this BMU were low concentrations of nitrates and dissolved inorganic nitrogen. In contrast, average concentrations of Chl *a* were higher than in other units. The lowest absolute AAP abundances were observed in this unit, however, bacterial production was the highest. The observed number of ASVs and Shannon index were high, with the maximum Pielou’s evenness recorded in all BMUs. At the genus level, *HIMB11* and *Nereida* from the family Rhodobacteraceae (Alphaproteobacteria) had lower values than in other units. Conversely, genera *Roseovarius*, *Puniceibacterium* and *CACIIZ01* from the family Rhodobacteraceae and the gammaproteobacterial *CABYJX01* (Halieaceae, Pseudomonadales) had maximum values in this unit.

BMU4 (8.64% of the data) was characterised by the lowest number of 7 samples. Seasonality was observed in this unit as almost all samples except one were from winter. A regional pattern was also noted, with stations towards the open sea predominating (CJ007 and CJ009). This unit was the shallowest one (average depth 27.5 m) and had the lowest average temperature of 14.7 °C and the lowest concentrations of nitrates and silicates. In contrast, Chl *a* was higher than the average of the other units. In terms of biotic variables, the absolute abundances of *Synechococcus* and HNF and the very high abundances of AAPs were pronounced in this unit, while the abundances of *Prochlorococcus* were minimum. At the genus level, *Planktomarina*, *CACIIZ01*, *Roseovarius* and *Planktotalea* from the Rhodobacteraceae family (Alphaproteobacteria) had the lowest mean values, while *CACIJG01*, *Rubricella*, *GCA2689605* (Hyphomicrobiaceae, Rhizobiales, Alphaproteobacteria) and *UBA868* (Arenicellales, Gammaproteobacteria) reached the highest values compared to the other units.

Unit BMU5 (34.57% of the data) collected the largest amount of data with 28 samples. Very high average salinity values (38.35) and temperatures above 16 °C together with the highest nitrites, silicates and Chl *a* defined this unit. Absolute record abundances of *Prochlorococcus* and picoeukaryotes, high abundances of *Synechococcus* and AAPs described this environment. Conversely, the observed number of ASVs in this unit was the lowest (88.32), as was Shannon diversity and Pielou’s evenness. Both gammaproteobacterial *Luminiphilus* and alphaproteobacterial *LFER01*, genera that dominated in all BMUs, had the highest mean scores in this unit. Although rarely found in other BMUs, *Roseobacter* and *Sphingomonas* reached higher values, while numerous genera such as *RS62, Rubricella*, *SP197* and *Erythrobacter* had the lowest ones.


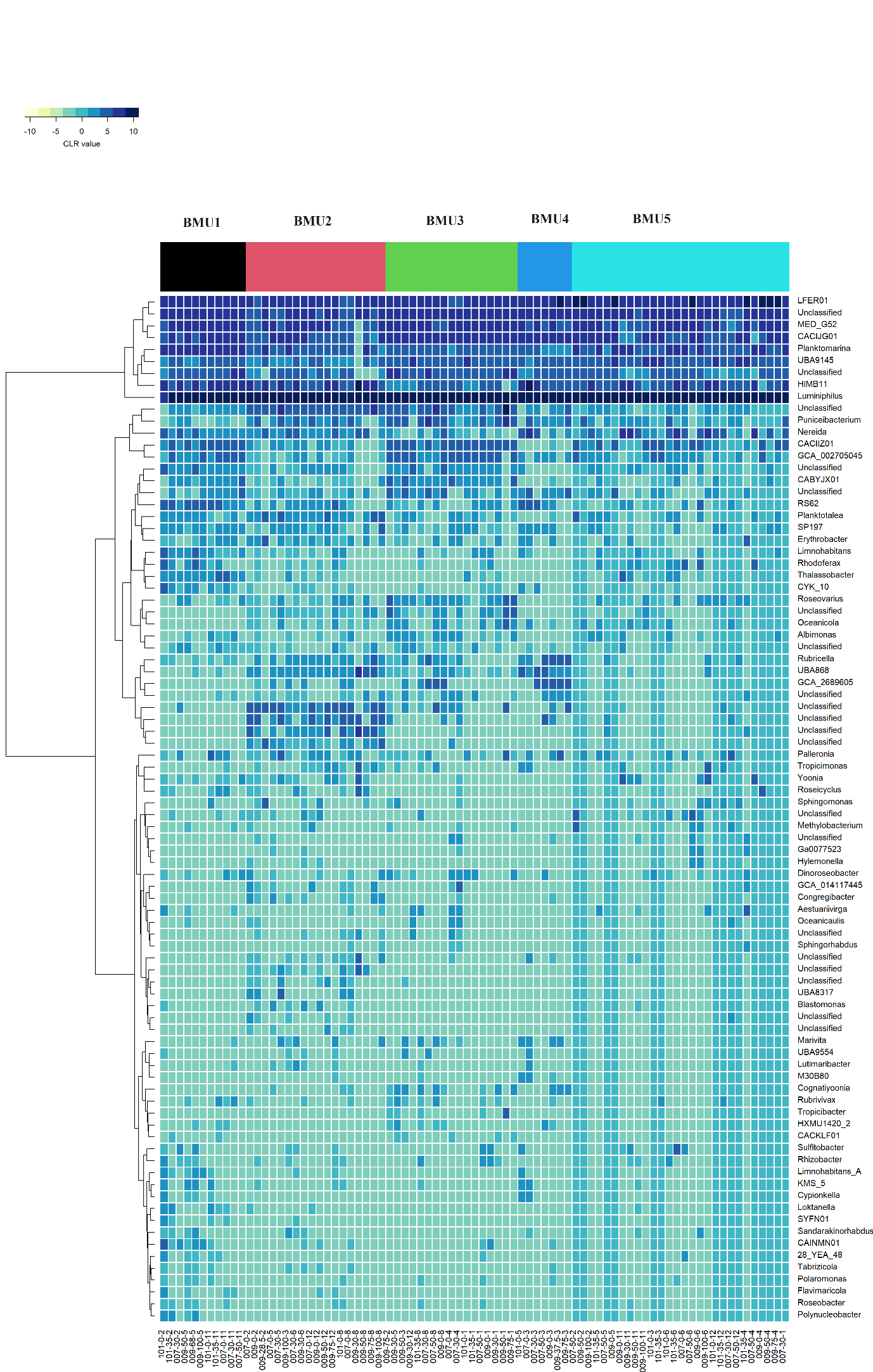


**Figure S2.** Genus-agglomerated and CLR-transformed *puf*M dataset resulting in five distinct BMUs. Samples are shown for each BMU.

**ASV-LEVEL** **NEURAL GAS ANALYSIS**

BMU1 had the highest average temperature (16.86) compared to other units with maximum average abundances of almost all biotic variables (highest total bacteria count, % of HNA bacteria, Synechococcus, Prochlorococcus, HNF and AAPs). Average concentrations of chl a were highest in this unit. On contrary, average ammonia concentrations and total phosphorous were the lowest. ASV232 (UBA868) had the highest average value in this unit.

BMU2 was the shallowest environment (average depth 29.04 m) described with lowest contribution of HNA bacteria, Prochlorococcus and lowest bacterial production. Concentrations of DIN and NTOT as well as silicate were the lowest. Salinity and total phosphorous were the highest in this unit. Noticeable were very low values of all ASVs belonging to genus CACIJG01 (Rhodobacteraceae) in this family. ASV515 (Methylobacterium, Rhizobiales order) and ASV66 (Nereida, Rhodobacteraceae) had lowest values compared to other units.

BMU3 could be described as nutrient-enriched environment (highest concentration of nitrate, ammonia, DIN, NTOT, orthophosphate and silicate) with the lowest total bacteria count, as well as lowest abundances of Synechococcus, picoeukaryots, HNF and AAPs. Salinity, nitrite and Chl a had the lowest values. ASVs with highest values in this unit compared to others were ASV206 (genus CABYJX01, Halieaceae family). ASV122 (genus CACIIZ01, Rhodobacteraceae family), ASV193 (GCA_002705045, Rhodobacteraceae family). ASVs (ASV18, ASV36, ASV39) of the genus CACIJG01 (Rhodobacteraceae family) dominated this unit compared to others.

Distinct factors that described BMU4 were highest counts of picoeukaryots and nitrite concentration. On contrary, bacterial production as well as concentrtions of nitrate, NTOT, orthophosphate and total phosphate were the lowest. This unit gathered mostly winter samples. Interesting to consider is that different ASVs (for example ASV122 and ASV386) within the genus CACIIZ01 had inverse values in this unit. Similary to BMU3, ASV122 was detected in higher values. ASV345 (genus Limnohabitans) had high value only in this unit.

BMU5 was the deepest unit with lowest average temperature and salinity as well as the highest bacterial produciton. Absolute average abundances of Synechococcus were high. Distinct ASV with highest average values compared to other units was ASV448 (UBA8317).


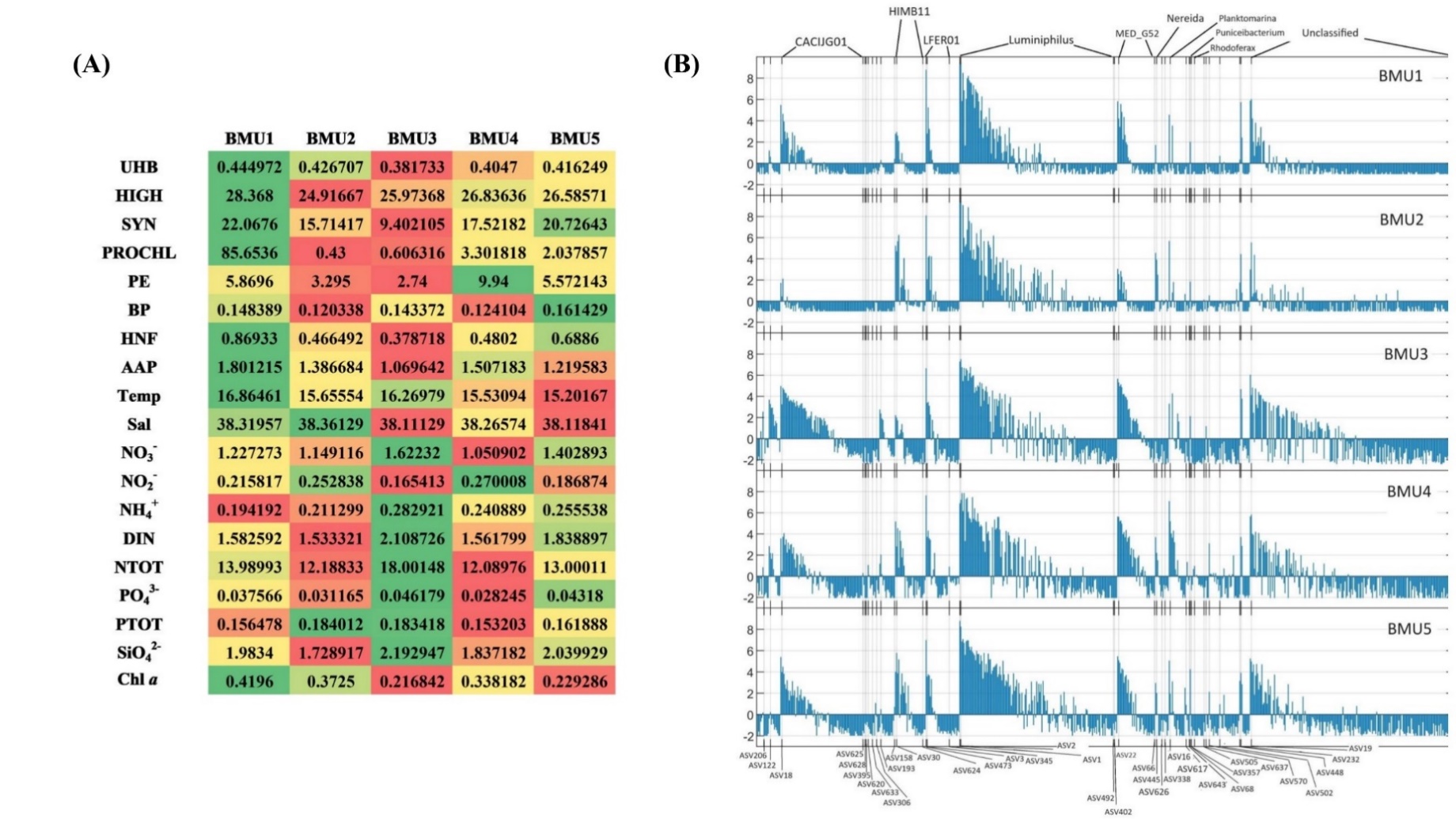


**Figure S3.** Neural gas analysis results of *puf*M dataset at clr-transformed ASV-level, clustered into five BMUs. Biotic (UHB, HIGH, SYN, PROCHL, PE, BP, HNF, AAP) and abiotic (Temp, Sal, NO_3_^-^, NO_2_^-^, NH_4_^+^, DIN, NTOT, PO_4_^3-^, PTOT, SiO_4_^2-^, Chl *a*) variables of each environment are given in (A) as average value for each unit. Colour gradient from red to green represents the lowest and highest average values respectively. Relative contribution expressed as CLR-transformed value of specific ASV is shown in (B). ASVs are ordered by genus, with most dominant genera ordered alphabetically.
